# TNF‐*α*‐Induced KAT2A Impedes BMMSC Quiescence by Mediating Succinylation of the Mitophagy‐Related Protein VCP

**DOI:** 10.1002/advs.202303388

**Published:** 2023-12-25

**Authors:** Zepeng Su, Jinteng Li, Jiajie Lin, Zhikun Li, Yunshu Che, Zhaoqiang Zhang, Guan Zheng, Guiwen Ye, Wenhui Yu, Yipeng Zeng, Peitao Xu, Xiaojun Xu, Zhongyu Xie, Yanfeng Wu, Huiyong Shen

**Affiliations:** ^1^ Department of Orthopedics The Eighth Affiliated Hospital of Sun Yat‐Sen University Shenzhen 518000 China; ^2^ Center for Biotherapy The Eighth Affiliated Hospital of Sun Yat‐Sen University Shenzhen 518000 China

**Keywords:** BMMSC, mitophagy, quiescence, succinylation, TNF‐*α*

## Abstract

Regular quiescence and activation are important for the function of bone marrow mesenchymal stem cells (BMMSC), multipotent stem cells that are widely used in the clinic due to their capabilities in tissue repair and inflammatory disease treatment. TNF‐*α* is previously reported to regulate BMMSC functions, including multilineage differentiation and immunoregulation. The present study demonstrates that TNF‐*α* impedes quiescence and promotes the activation of BMMSC in vitro and in vivo. Mechanistically, the TNF‐*α*‐induced expression of KAT2A promotes the succinylation of VCP at K658, which inhibits the interaction between VCP and MFN1 and thus inhibits mitophagy. Furthermore, activated BMMSC exhibits stronger fracture repair and immunoregulation functions in vivo. This study contributes to a better understanding of the mechanisms of BMMSC quiescence and activation and to improving the effectiveness of BMMSC in clinical applications.

## Graphical abstract

TNF‐*α* enhances the functionality of bone marrow mesenchymal stem cells (BMMSC) by impeding the quiescence and promoting the activation of BMMSC. Mechanistically, TNF‐*α* inhibits mitophagy and promotes OXPHOS via KAT2A‐manipulated succinylation of VCP‐K658, bringing about the activation of BMMSC. This study contributes to improving the utility of BMMSC in clinical applications.

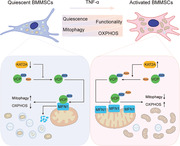

## 1 Introduction

Bone marrow mesenchymal stem cells (BMMSC) are pluripotent stem cells that are widely used in the clinic due to their powerful immunoregulation and multilineage differentiation capabilities.^[^ ^1^ ^]^ Under in vivo physiological conditions, BMMSC mainly remains in a quiescent state, a reversible cellular state of arrest in the G0 phase, to preserve key functional features.^[^ ^2^ ^]^ These quiescent MSCs are activated rapidly in response to external stimuli, which permits them to exhibit functions in tissue repair and immunotherapy.^[^ ^1^, ^3^ ^]^ This process is under the control of various cytokines, including TNF‐*α*, which have been identified as a key regulator of BMMSC functions.^[^ ^4^ ^]^ Clarifying the role of TNF‐*α* in quiescence as well as the detailed mechanisms underlying its activity will contribute to improving the effectiveness of BMMSC in clinical applications.

Stem cells undergo a shift in energy metabolism from quiescence to activation.^[^ ^5^ ^]^ As intracellular energy supply stations, mitochondria play an important role in regulating cell energy metabolism by altering their number and function.^[^ ^6^ ^]^ Mitophagy is a selective type of autophagy that degrades mitochondria and thus decreases the number of mitochondria to maintain low metabolic levels.^[^ ^6^, ^7^ ^]^ Recent studies have demonstrated that mitophagy is one of the critical mechanisms regulating the quiescence of stem cells.^[^ ^8^ ^]^ VCP is a regulatory protein that promotes mitophagy through different mechanisms, including by recruiting LC3B to mitochondria or removing MFN1/2 from mitochondria.^[^ ^9^ ^]^ Nevertheless, whether VCP‐regulated mitophagy is involved in the regulation of BMMSC quiescence remains unknown.

Succinylation is a newly discovered protein modification that can regulate protein localization, stability, structure, and function.^[^ ^10^ ^]^ Protein succinylation reportedly inhibits mitophagy and impairs mitochondrial respiration and metabolic inflexibility.^[^ ^11^ ^]^ A previous study showed that hypersuccinylation promotes the proliferation of BMMSC through metabolic switching.^[^ ^12^ ^]^ However, whether protein succinylation participates in regulating mitophagy and the subsequent quiescence of BMMSC remains to be elucidated. KAT2A was recently determined to be a key protein in catalyzing succinylation.^[^ ^13^ ^]^ Furthermore, KAT2A is reportedly able to regulate the quiescent state of Hematopoietic stem cells (HSC).^[^ ^14^ ^]^ However, whether KAT2A‐mediated succinylation modification is involved in regulating BMMSC quiescence remains unreported.

The present study demonstrated that TNF‐*α* impeded BMMSC quiescence by inhibiting mitophagy. Mechanistically, the TNF‐*α*‐induced expression of KAT2A promoted the succinylation of VCP at K658, which blocked the interaction of VCP and MFN1 and thus inhibited mitophagy. Furthermore, in vivo experiments showed that activated BMMSC exhibited stronger bone defect repair capacity and were better able to relieve arthritis symptoms than quiescent BMMSC. This study further elucidates the mechanism of BMMSC quiescence and provides new insight and targets for improving the effectiveness of BMMSC in clinical applications.

## 2 Results

### 2.1 The Phenotypes and Differentiation Potentials of BMMSC

BMMSCs were isolated from human and mouse bone marrow and expanded in vitro. The phenotypes of human and mouse BMMSC were validated by flow cytometry, and their differentiation potentials were detected by Alizarin Red S (ARS), Oil Red O, and Alcian Blue staining (Figure S1a–d, Supporting Information). In addition, the differentiation potentials of the genetically modified BMMSC used in this study were also detected (Figure S1e,f, Supporting Information)

### 2.2 TNF‐*α* Impedes the Quiescence and Promotes the Activation of BMMSC

**Figure 1:**
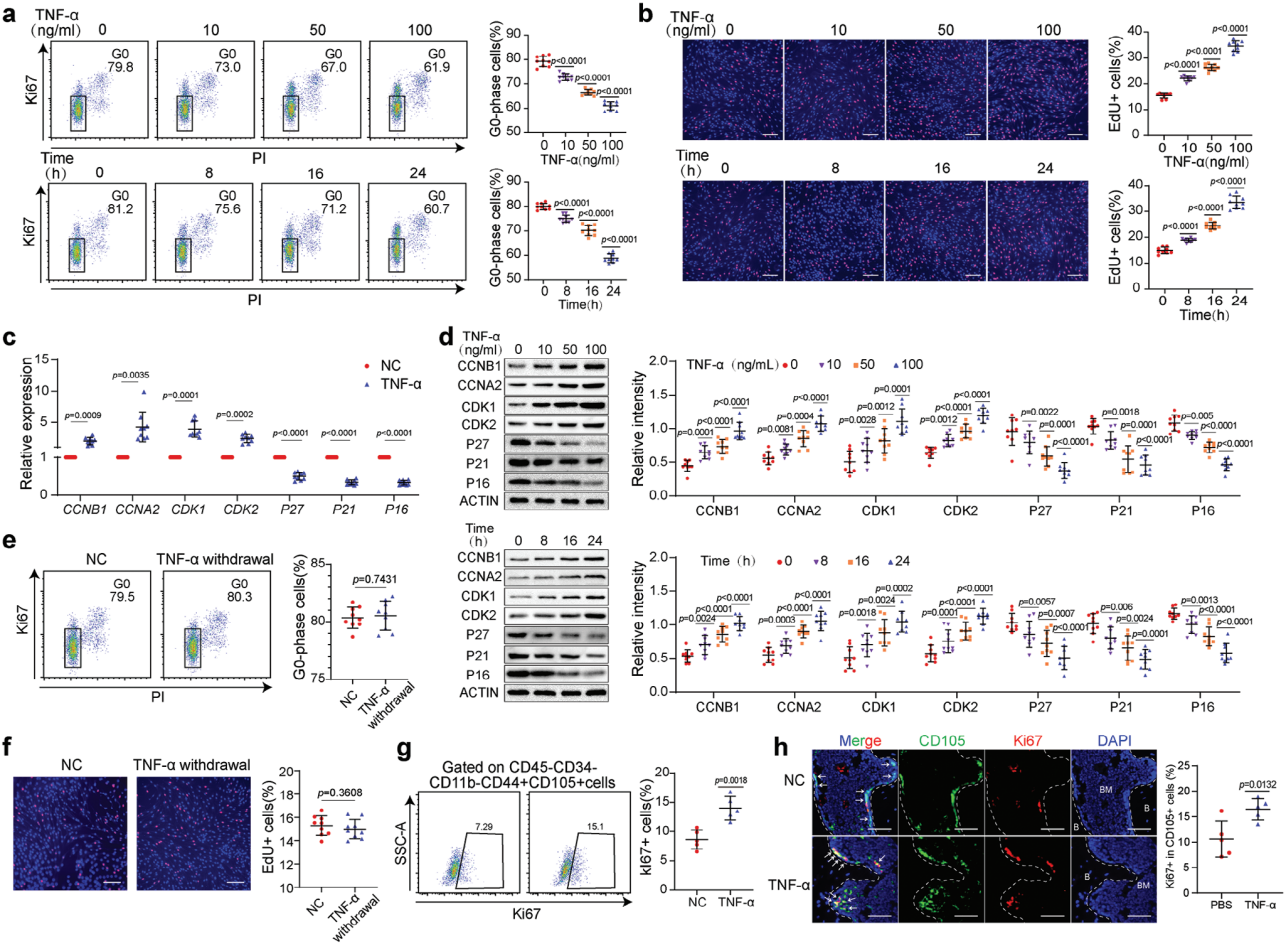
TNF‐*α* impedes the quiescence and promotes the activation of BMMSC. a) TNF‐*α* decreased the proportion of G0‐phase human BMMSC (hBMMSC) (*n* = 9) in concentration‐ and time‐dependent manners. b) TNF‐*α* increased the proportion of EdU+ hBMMSC (*n* = 9) in concentration‐ and time‐dependent manners. Scale bar = 200 µm. c) TNF‐*α* altered the mRNA levels of cell cycle‐related genes in hBMMSC (*n* = 9). d) TNF‐*α* altered the protein levels of cell cycle‐related genes in hBMMSC (*n* = 9) in concentration (top)‐ and time (bottom)‐dependent manners. e, f) After the withdrawal of TNF‐*α*, the proportions of G0‐phase (e) and EdU+ (f) hBMMSC (*n* = 9) were equal to the NC group. Scale bar = 200 µm. g, h Flow cytometry analysis g) and immunofluorescence assays h) showed that TNF‐*α* increased the proportion of Ki67+ BMMSC (*n* = 5) in vivo. The dotted line represents the demarcation between the bone marrow and trabeculae. The arrows indicate BMMSC, B indicates bone, and BM indicates the bone marrow. Scale bar = 50 µm. The values are presented as the mean ± SD. The statistical analyses were performed as follows: one‐way ANOVA followed by Bonferroni's post hoc comparisons (a, b, d), two‐tailed paired t‐test (c, e, f), and two‐tailed Student's t‐test (g, h).

Treatment with TNF‐*α* reduced the proportion of G0‐phase BMMSC, representing quiescent BMMSC, with increases in the TNF‐*α* concentration and treatment duration (**Figure** **1**a). In addition, an increase in the proportion of EdU+ BMMSC, which represents activated BMMSC, was observed with increases in the concentration and duration of TNF‐*α* treatment (Figure 1b). Consistently, increases in the expression of the cell cycle‐related factors CCNB1, CCNA2, CDK1, and CDK2, and decreases in the expression of P16, P21, and P27 were observed in TNF‐*α*‐treated BMMSC at both the mRNA and protein levels (Figure 1c,d). The above effects were dependent on both the TNF‐*α* concentration and the treatment duration. In addition, the effects of higher concentrations of TNF‐*α* were also explored and a more prominent effect was not observed, suggesting that 100 ng mL^−1^ is a saturating concentration to activate BMMSC (Figure S2a,b, Supporting Information), and 100 ng mL^−1^ TNF‐*α* treatment for 24 h was adopted for the follow‐up experiments. To explore whether the effects of TNF‐*α* were reversible, TNF‐*α* was withdrawn after 24 h treatment. There was no significant difference in either the proportion of G0‐phase BMMSC or the proportion of EdU+ BMMSC between the TNF‐*α* withdrawal group and the control group (Figure 1e,f), indicating that the effects of TNF‐*α* were reversible. To explore the role of TNF‐*α* in regulating BMMSC quiescence in vivo, TNF‐*α* was injected locally into the femurs of mice for three consecutive days. Flow cytometry analysis was performed and the gating strategies were presented in Figure S3 (Supporting Information). The results showed that TNF‐*α* increased the proportion of Ki67+ BMMSC in vivo (Figure 1g), and this finding was further confirmed by immunofluorescence assay (Figure 1h). These results suggest that TNF‐*α* impedes the quiescence and promotes the activation of BMMSC in vitro and in vivo.

### 2.3 TNF‐*α* Impedes BMMSC Quiescence by Inhibiting Mitophagy

**Figure 2:**
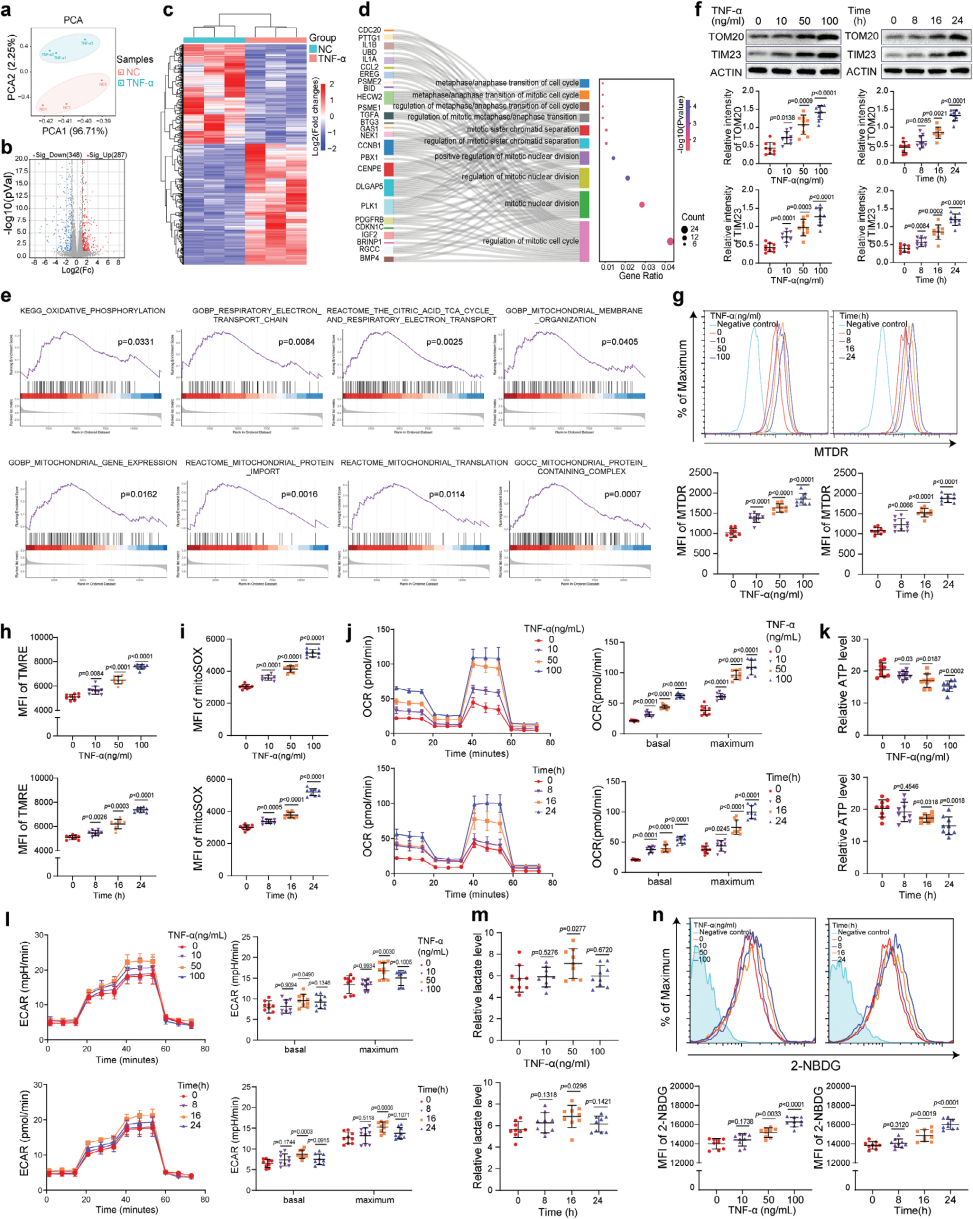
TNF‐*α* elevates the number of mitochondria and promotes OXPHOS activity in BMMSC. a) The PCA of hBMMSC treated with (*n* = 3) and without (*n* = 3). b) Volcano plot of hBMMSC treated with (*n* = 3) and without (*n* = 3) TNF‐*α*. c) The cluster heatmap of hBMMSC treated with (*n* = 3) and without (*n* = 3) TNF‐*α*. d) The GO‐BP analysis of differentially expressed genes in hBMMSC (*n* = 3) treated with and without TNF‐*α*. e) The GSEA of hBMMSC treated with (*n* = 3) and without (*n* = 3) TNF‐*α*. f) TNF‐*α* increased the protein levels of TOM20 and TIM23 in BMMSC (*n* = 9) in concentration‐ and time‐dependent manners. g–j) TNF‐*α* increased the MFI of MTDR (g), TMRE (h), and MitoSOX (i), and the OCR levels (j) in hBMMSC (*n* = 9) in concentration‐ and time‐dependent manners. k) TNF‐*α* decreased the ATP level of hBMMSC (*n* = 9) in a concentration‐ and time‐dependent manner. l, m) Alterations in the ECAR (l) and Lactate (m) levels in hBMMSC (*n* = 9) after treatment with concentration and time gradient of TNF‐*α*. n TNF‐*α* promoted glucose uptake of hBMMSC (*n* = 9) in concentration‐ and time‐dependent manners. The values are presented as the mean ± SD. The statistical analyses were performed with one‐way ANOVA followed by Bonferroni's post hoc comparisons.

To explore the mechanism by which TNF‐*α* impedes BMMSC quiescence, a transcriptome sequencing of BMMSC treated with or without TNF‐*α* was performed. The principal component analysis (PCA) revealed that TNF‐*α* significantly altered the transcriptome of BMMSC (**Figure** **2** **a**). The heatmap and volcano plot showed a total of 635 differentially expressed genes, including 348 upregulated genes and 287 downregulated genes after TNF‐*α* treatment (Figure 2b,c). The GO‐BP analysis revealed the enrichment of multiple cell cycle‐related pathways, further confirming the effect of TNF‐*α* on BMMSC quiescence (Figure 2d).

The gene set enrichment analysis (GSEA) revealed that the functional state of mitochondria was significantly altered by TNF‐*α* treatment (Figure 2e). The expression levels of the

mitochondrial outer membrane protein TOM20 and the inner membrane protein TIM23 were significantly increased after TNF‐*α* treatment (Figure 2f). Consistently, the flow cytometry analysis also revealed that the mean fluorescence intensity (MFI) of MitoTracker Deep Red (MTDR) was significantly elevated in TNF‐*α*‐activated BMMSC, indicating that TNF‐*α* increased the number of mitochondria in BMMSC (Figure 2g). After treatment with TNF‐*α*, elevated MFI of TMRE and MitoSOX were also detected, which hinted at an increase in mitochondrial OXPHOS activity (Figure 2h,i). As important indexes of OXPHOS, both the basal and maximum oxygen consumption rate (OCR) were increased by TNF‐*α* in both concentration‐ and time‐dependent manners, indicating that TNF‐*α* enhanced the OXPHOS activity of BMMSC (Figure 2j). However, the ATP level decreased after treatment with TNF‐*α*, potentially due to an increase in ATP consumption after BMMSC exited quiescence and entered the cell cycle (Figure 2k). As an important energy metabolism process, the levels of glycolysis were also measured. Treatment with 50 ng mL^−1^ TNF‐*α* for 24 h significantly increased the extracellular acidification rate (ECAR) and lactate production, whereas treatment with 10 or 100 ng mL^−1^ TNF‐*α* for 24 h had no such effects (Figure 2l,m). A similar phenomenon was observed after treatment of BMMSC with a time gradient of TNF‐*α* (Figure 2l,m). However, the glucose consumption levels were elevated by TNF‐*α* in both concentration‐ and time‐dependent manners, which might be related to the TNF‐*α*‐enhanced OXPHOS which required a large amount of glucose (Figure 2n). These results suggest that mitochondrial OXPHOS rather than glycolysis may be involved in the TNF‐*α*‐induced activation of BMMSC.

**Figure 3:**
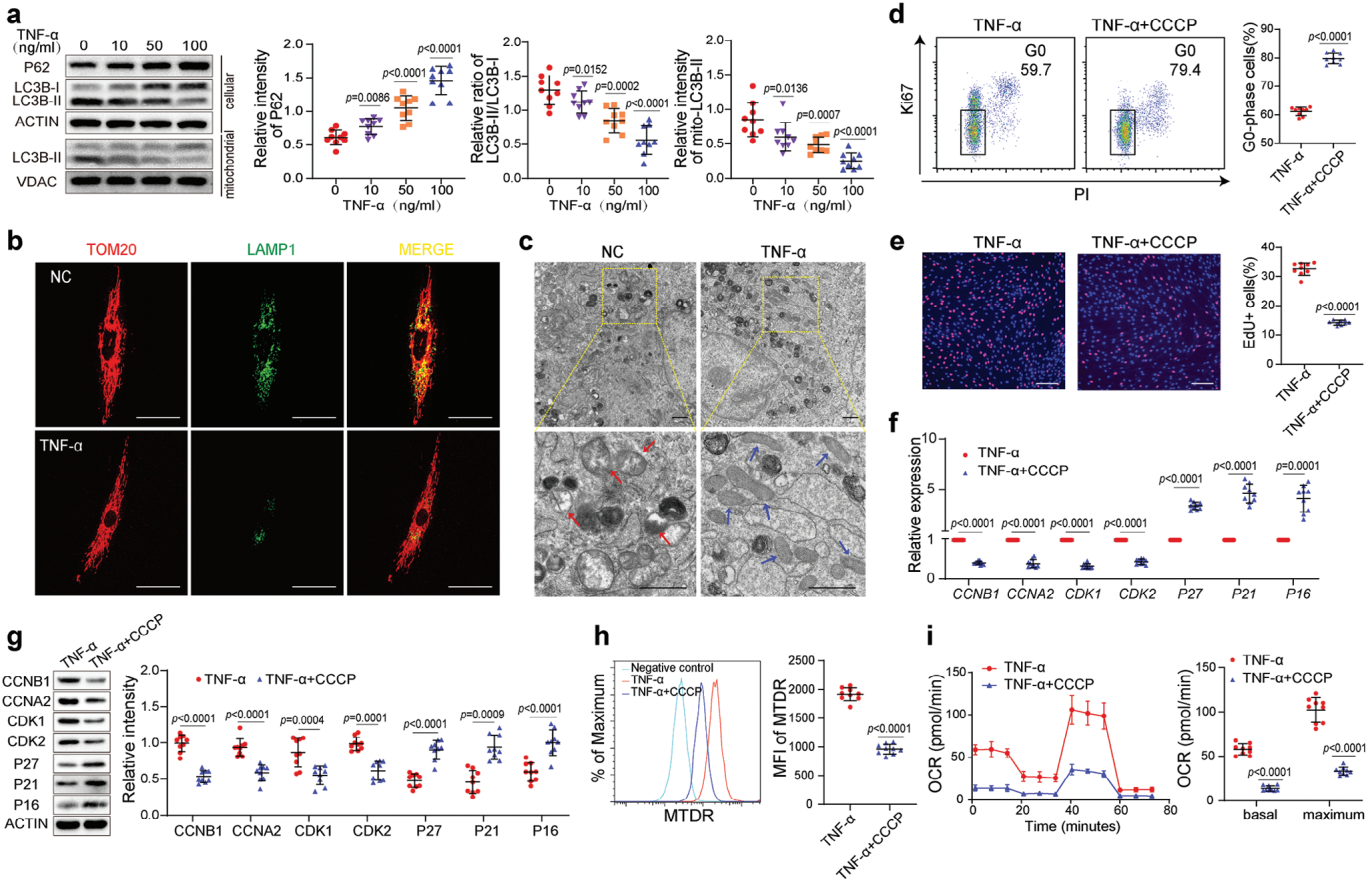
TNF‐*α* impedes BMMSC quiescence by inhibiting mitophagy. a) TNF‐*α* increased P62 level and decreased LC3B‐II/LC3B‐I ratio and mitochondria‐related LC3B‐II level in hBMMSC (*n* = 9) in concentration‐dependent manners. b) TNF‐*α* inhibited the colocalization (yellow) of mitochondria (red) and lysosomes (green) in hBMMSC. Scale bar = 50 µm. c) TEM showed fragmented mitochondria and mitochondria engulfed by autophagosomes in hBMMSC treated without TNF‐*α* and a larger number of healthy mitochondria in hBMMSC treated with TNF‐*α*. Scale bar = 2 µm. d, e) CCCP (5 µm) increased the proportion of G0‐phase hBMMSC (d) and decreased the proportion of EdU+ hBMMSC (e) (*n* = 9) in the presence of TNF‐*α*. Scale bar = 200 µm. f, g) CCCP altered the mRNA (f) and protein (g) levels of cell cycle‐related genes in hBMMSC (*n* = 9) in the presence of TNF‐*α*. h, i CCCP reduced the MTDR MFI h) and the OCR i) of hBMMSC (*n* = 9) in the presence of TNF‐*α*. The values are presented as the mean ± SD. The statistical analyses were performed as follows: one‐way ANOVA followed by Bonferroni's post hoc comparisons (a), and two‐tailed paired t‐test (d–i).

The number of mitochondria is mainly regulated through mitophagy.^[^ ^6b^ ^]^ Herein, the protein level of P62 was increased and the LC3B‐II/LC3B‐I ratio was decreased in TNF‐*α*‐activated BMMSC (**Figure** **3**a). Furthermore, the level of mitochondria‐associated LC3B‐II (LC3B‐II in mitochondria) was decreased after TNF‐*α* treatment (Figure 3a). Autophagic mitochondria are phagocytosed and degraded by lysosomes.^[^ ^7^ ^]^ By fluorescently labeling the mitochondrial protein TOM20 and the lysosomal protein LAMP1, we found that TNF‐*α* inhibited the colocalization of mitochondria with lysosomes (Figure 3b). Transmission electron microscopy (TEM) showed that TNF‐*α* attenuated mitochondrial fragmentation and reduced mitochondria

engulfed by autophagosomes, resulting in the retainment of a larger number of healthy mitochondria (Figure 3c). Furthermore, CCCP, an inducer of mitophagy, blocked the inhibitory effect of TNF‐*α* on BMMSC quiescence (Figure 3d). Similarly, the TNF‐*α*‐increased proportion of EdU+ BMMSC was decreased by CCCP (Figure 3e). At both mRNA and protein levels, the expression of CCNB1, CCNA2, CDK1, and CDK2 was decreased and that of P16, P21, and P27 was increased by CCCP in the presence of TNF‐*α* (Figure 3f,g). Furthermore, the elevated MTDR MFI and OCR induced by TNF‐*α* were also reduced by CCCP (Figure 3h,i). These results suggest that TNF‐*α* inhibits mitophagy to increase the number of mitochondria and enhance OXPHOS activity, which then impedes the quiescence and promotes the activation of BMMSC.

### 2.4 Succinylation of the Mitophagy‐Related Protein VCP is Involved in the TNF‐*α*‐Regulated Quiescence of BMMSC

**Figure 4:**
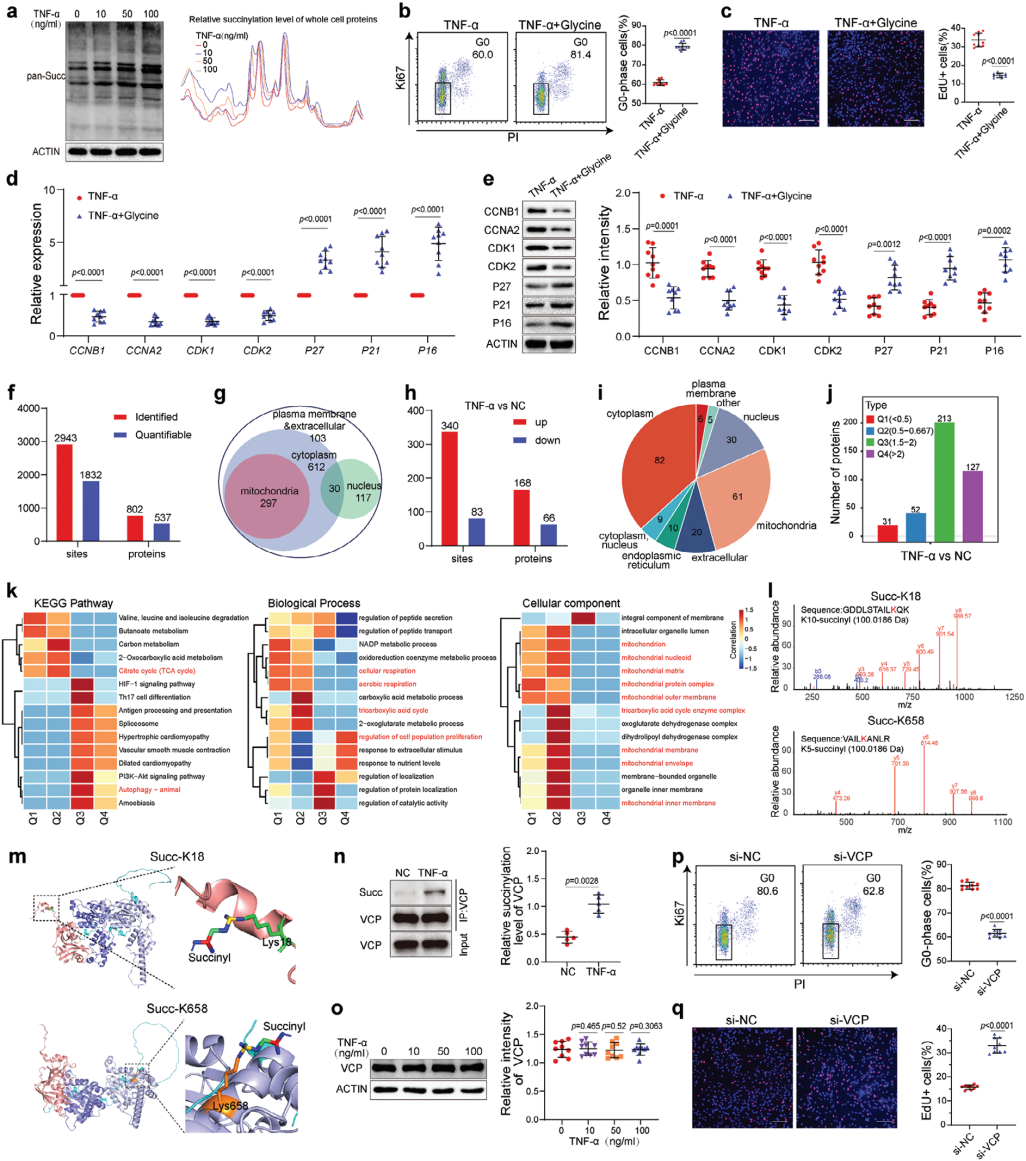
Succinylation of the mitophagy‐related protein VCP is involved in the TNF‐*α*‐regulated quiescence of BMMSC. a) TNF‐*α* elevated the protein succinylation level in hBMMSC (*n* = 9) in a concentration‐dependent manner. b, c) Treatment with 100 mm glycine increased the proportion of G0‐phase hBMMSC (b) and decreased the proportion of EdU+ hBMMSC (c) (*n* = 9) in the presence of TNF‐*α*. Scale bar = 200 µm. d, e) Glycine altered the mRNA (d) and protein (e) levels of cell cycle‐related genes in hBMMSC (*n* = 9) in the presence of TNF‐*α*. f) Histogram showing the number of succinylation sites and the corresponding proteins detected in hBMMSC. g) Venn diagram showing the distribution of succinylated proteins in hBMMSC. h) Histogram showing the number of TNF‐*α*‐regulated succinylation sites and the corresponding proteins. i) Pie chart showing the distribution of proteins with TNF‐*α*‐regulated succinylation. j) The TNF‐*α*‐regulated succinylation sites were divided into Q1–Q4 groups according to the fold change in the succinylation level. k) KEGG, GO‐BP, and GO‐CC analyses of proteins in groups Q1–Q4. l) Succinylation profiling identified K18 and K658 as succinylation sites of VCP. m) Pattern diagram of the succinylation sites of VCP. The salmon color indicates the N‐terminal domain, the slate blue color indicates the D1 ATPase domain and the light blue color indicates the D2 ATPase domain. n) Immunoprecipitation followed by western blotting verified that TNF‐*α* elevated the succinylation level of VCP in hBMMSC (*n* = 5). o) The protein level of VCP was not altered by TNF‐*α* (*n* = 9). p, q) The knockdown of VCP decreased the proportion of G0‐phase hBMMSC (p) and increased the proportion of EdU+ hBMMSC (q) (*n* = 9) in the absence of TNF‐*α*. Scale bar = 200 µm. The values are presented as the mean±SD. The statistical analyses were performed as follows: one‐way ANOVA (o), two‐tailed paired t‐test (c, e, f), and two‐tailed Student's t‐test (b‐e, n, p, q).

Protein succinylation reportedly plays an essential role in regulating mitophagy.^[^ ^11^ ^]^ Therefore, we sought to determine whether protein succinylation participated in regulating mitophagy and quiescence in BMMSC. To this end, we first examined the succinylation level of the BMMSC proteome under TNF‐*α* treatment. Compared with the control group, TNF‐*α*‐treated BMMSC showed an increased level of proteome succinylation (**Figure** **4**a). Treatment with glycine, an inhibitor of protein succinylation, reduced the protein succinylation level of BMMSC (Figure S4a, Supporting Information). In addition, glycine increased the proportion of G0‐phase BMMSC and decreased the proportion of EdU+ BMMSC in the presence of TNF‐*α* (Figure 4b,c). The expression of CCNB1, CCNA2, CDK1, and CDK2 was downregulated and that of P16, P21, and P27 was upregulated in BMMSC treated with glycine (Figure 4d,e). Furthermore, glycine treatment decreased the protein levels of TOM20 and TIM23 as well as the MTDR MFI (Figure S4b,c, Supporting Information). The protein level of P62 was decreased, and the LC3B‐II /LC3B‐I ratio and mitochondria‐associated LC3B‐II were increased, after treatment with glycine (Figure S4b, Supporting Information). Glycine also promoted the colocalization of mitochondria with lysosomes, enhanced mitochondrial fragmentation, and increased the number of mitochondria engulfed by autophagosomes (Figure S4d,e, Supporting Information). In addition, the MFI of TMRE and MitoSOX and the OCR were decreased, and ATP levels were increased in BMMSC treated with glycine (Figure S4f–i, Supporting Information). These results suggest that TNF‐*α* inhibits mitophagy and impedes BMMSC quiescence by regulating protein succinylation.

To further explore the mechanisms by which TNF‐*α* regulates mitophagy to impede BMMSC quiescence, the protein succinylation of BMMSC treated with or without TNF‐*α* was analyzed. A total of 2943 succinylation sites in 802 proteins were identified, of which 1832 sites and 537 proteins were quantifiable (Figure 4f). A total of 297 succinylated proteins were localized to mitochondria (Figure 4g). TNF‐*α* regulated the succinylation levels of 234 proteins at 423 sites (fold change>1.5), of which 340 exhibited increased succinylation and 83 exhibited decreased succinylation, within 168 and 66 proteins, respectively (Figure 4h). Of the 234 proteins, 61 were localized to mitochondria (Figure 4i). The 423 sites were divided into four groups (Q1, Q2, Q3, and Q4) according to the fold change in their succinylation levels after TNF‐*α* treatment (Figure 4j). KEGG and GO‐BP analyses of the proteins in Q1–Q4 groups revealed enrichments of autophagy and multiple mitochondrial metabolism‐related pathways, and the GO‐CC analysis revealed enrichments of several mitochondrial component pathways, suggesting that succinylation plays an important role in regulating mitochondrial metabolism and function (Figure 4k). Of the proteins whose succinylation levels were regulated by TNF‐*α*, we noted a highly expressed ATPase, VCP, which plays a regulatory role in the cell cycle and mitophagy in stem cells.^[^ ^9^, ^15^ ^]^ The succinylation levels at two sites of VCP (K18, located in the N‐terminal domain, and K658, located in the D2 ATPase domain) were upregulated after TNF‐*α* treatment (Figure 4l,m). By immunoprecipitation and western blotting, the elevated succinylation level of VCP induced by TNF‐*α* was verified (Figure 4n). Although the protein level of VCP was not altered by TNF‐*α* (Figure 4o), the knockdown of VCP significantly decreased the proportion of G0‐phase BMMSC and increased the proportion of EdU+ BMMSC (Figure 4p,q). These results suggest that the succinylation level of the mitophagy‐related protein VCP may be involved in the regulatory effects of TNF‐*α* on BMMSC quiescence.

### 2.5 The Succinylation of VCP at K658 Rather Than K18 Contributes to Regulating Mitophagy and the Subsequent Quiescence of BMMSC

**Figure 5:**
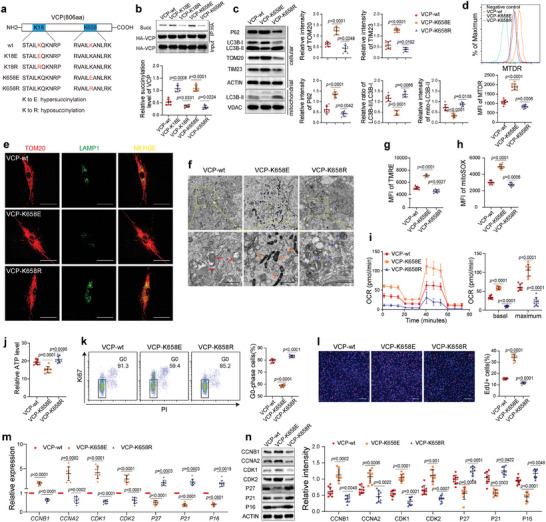
Succinylation of VCP at K658 contributes to regulating mitophagy and the subsequent quiescence of BMMSC. a) Schematic diagram of the construction of VCP mutants. b) Compared with VCP‐Wt, VCP‐K18E and VCP‐K658E increased the succinylation level of VCP, while VCP‐18R and VCP‐K658R decreased the succinylation level of VCP (*n* = 5). c) Compared with VCP‐Wt, VCP‐K658E upregulated the protein levels of TOM20, TIM23 and P62 and reduced LC3B‐II/LC3B‐I ratio and mitochondria‐related LC3B‐II level in hBMMSC (*n* = 9). VCP‐K658R induced the opposite changes. d) Compared with VCP‐Wt, VCP‐K658E and K658R elevated and reduced the MFI of MTDR in hBMMSC (*n* = 9), respectively. e) Compared with VCP‐Wt, VCP‐K658E, and VCP‐K658R decreased and increased the colocalization of mitochondria and lysosomes, respectively. Scale bar = 50 µm. f) Compared with VCP‐Wt, VCP‐K658E decreased the numbers of fragmented mitochondria and mitochondria engulfed by autophagosomes, whereas VCP‐K658R exhibited the opposite effect. Scale bar = 2 µm. g–i) Compared with VCP‐Wt, VCP‐K658E increased the MFI of TMRE (g) and MitoSOX (h) and the OCR (i) in hBMMSC (*n* = 9), whereas VCP‐K658R exhibited the opposite effect. j Compared with VCP‐Wt, VCP‐K658E, and VCP‐K658R decreased and increased the ATP levels in hBMMSC, respectively (*n* = 9). k, l) Compared with VCP‐Wt, VCP‐K658E decreased the proportions of G0‐phase hBMMSC (*n* = 9) (k) and increased the proportions of EdU+ hBMMSC (*n* = 9) (l). VCP‐K658R induced the opposite changes. Scale bar = 200 µm. m, n) Compared with VCP‐Wt, VCP‐K658E and VCP‐K658R had opposite effects on the mRNA (m) and protein (n) levels of cell cycle‐related genes in hBMMSC (*n* = 9). The values are presented as the mean ± SD. The statistical analyses were performed with one‐way ANOVA followed by Bonferroni's post hoc comparisons (b–d, g–n).

To further explore the succinylated sites of VCP in detail, the mutant proteins VCP‐K18E, VCP‐K18R, VCP‐K658E, and VCP‐K658R were constructed (**Figure** **5**a). VCP‐K18E and VCP‐K658E upregulated the succinylation level of VCP, whereas VCP‐K18R and VCP‐K658R downregulated the succinylation level of VCP (Figure 5b). Compared with wild‐type VCP (VCP‐Wt), VCP‐K658E increased the protein levels of TOM20 and TIM23 as well as the MFI of MTDR (Figure 5c,d). In addition, an increase in the P62 level and decreases in the LC3B‐II/LC3B‐I ratio and mitochondria‐associated LC3B‐II level were detected in BMMSC overexpressing VCP‐K658E (Figure 5c). Consistent with these observations, the colocalization of mitochondria and lysosomes was inhibited, and fragmented and autophagocytosed mitochondria were less abundant in BMMSC overexpressing VCP‐K658E (Figure 5e,f). The MFI of TMRE and MitoSOX and OCR were elevated, and ATP levels were reduced in BMMSC overexpressing VCP‐K658E compared with BMMSC overexpressing VCP‐Wt (Figure 5g–j). Furthermore, VCP‐K658R exhibited the opposite effects of VCP‐K658E (Figure 5c–j). However, VCP‐K18E and VCP‐K18R had no significant regulatory impact on mitophagy or OXPHOS (Figure S5a–c, Supporting Information). These results suggest that the succinylation of VCP‐K658 rather than VCP‐K18 regulates mitophagy and OXPHOS activity in BMMSC.

We then sought to determine the effect of the succinylation levels of the two sites of VCP on BMMSC quiescence. Flow cytometry analysis showed that VCP‐K658E significantly reduced the proportion of G0‐phase BMMSC compared with VCP‐Wt (Figure 5k). VCP‐K658E also increased the proportion of EdU+ BMMSC (Figure 5). Additionally, the expression of CCNB1, CCNA2, CDK1, and CDK2 was upregulated, and the expression of P16, P21, and P27 was downregulated in BMMSC overexpressing VCP‐K658E compared to BMMSC overexpressing VCP‐Wt (Figure 5m,n). The opposite effects of the above were observed in BMMSC overexpressing VCP‐K658R (Figure 5k–n). Furthermore, no significant difference in the proportion of G0‐phase BMMSC or EdU+ BMMSC was observed among BMMSC overexpressing VCP‐Wt, VCP‐K18E, and VCP‐K18R (Figure S5d,e, Supporting Information). These results suggest that the succinylation modification of VCP at K658 rather than K18 is involved in regulating mitophagy to control BMMSC quiescence.

### 2.6 The Succinylation of K658 Inhibits the Interaction Between VCP and MFN1

**Figure 6:**
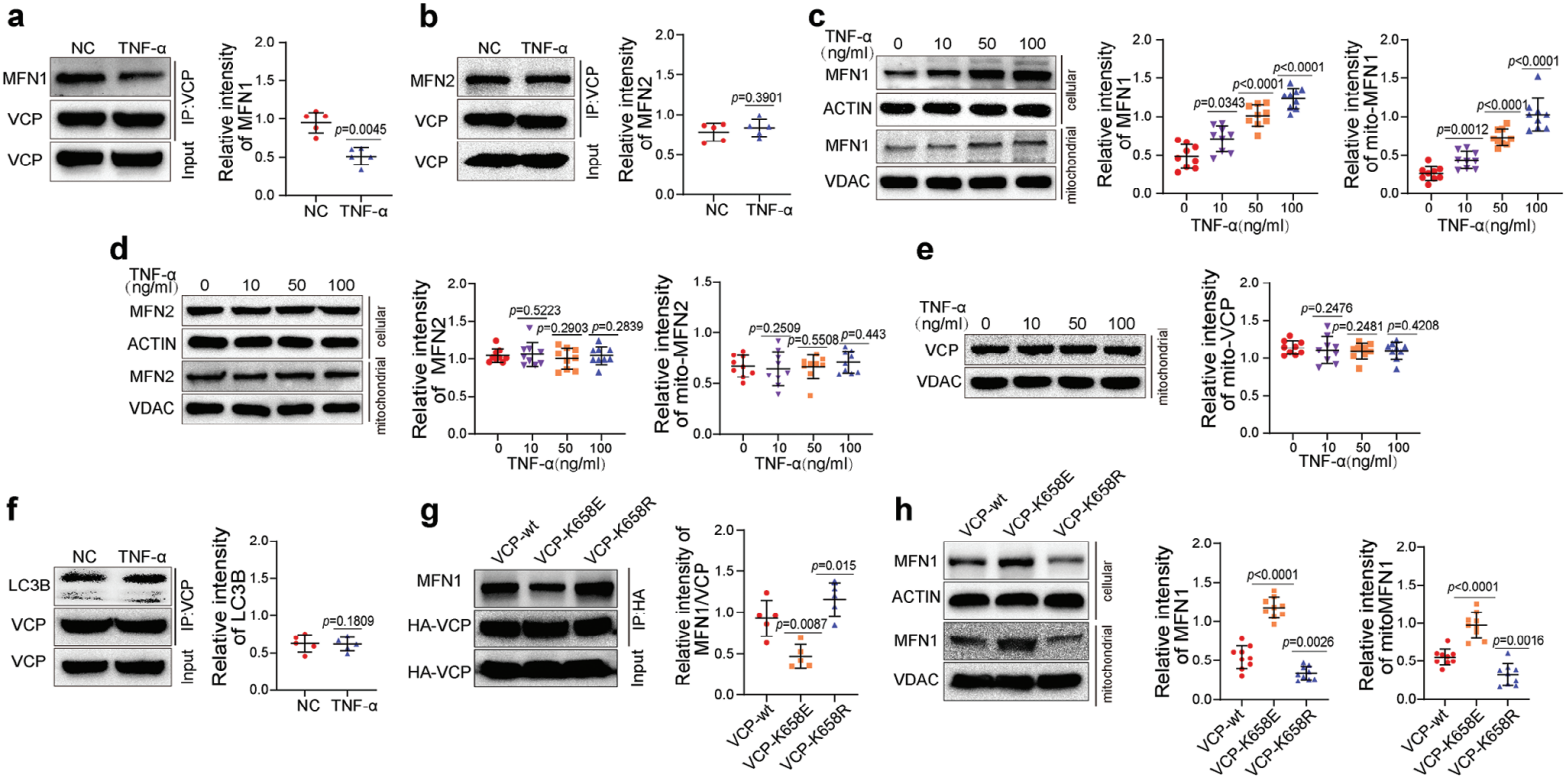
Succinylation of K658 inhibits the interaction of VCP and MFN1. a) TNF‐*α* inhibited the interaction of VCP and MFN1 in hBMMSC (*n* = 5). b) The interaction of VCP and MFN2 in hBMMSC (*n* = 5) was not altered by TNF‐*α*. c) TNF‐*α* upregulated the cellular and mitochondrial levels of MFN1 in hBMMSC (*n* = 9) in a concentration‐dependent manner. d) The cellular and mitochondrial levels of MFN2 in hBMMSC (*n* = 9) were not altered by TNF‐*α*. e) The mitochondrial levels of VCP in hBMMSC (*n* = 9) were not altered by TNF‐*α*. f) The interaction of VCP and LC3B in hBMMSC (*n* = 5) was not altered by TNF‐*α*. g) Compared with VCP‐Wt, VCP‐K658E and VCP‐K658R inhibited and promoted the interaction of VCP and MFN1 in hBMMSC (*n* = 5), respectively. h) Compared with VCP‐Wt, VCP‐K658E and VCP‐K658R upregulated and downregulated the cellular and mitochondrial levels of MFN1 in hBMMSC (*n* = 9), respectively. The values are presented as the mean ± SD. The statistical analyses were performed as follows: one‐way ANOVA followed by Bonferroni's post hoc comparisons (c–e, g, h) and two‐tailed paired t‐test (a, b, f).

Previous studies revealed that VCP promotes mitophagy by binding to MFN1 and MFN2, drawing them away from the mitochondria and inhibiting mitochondrial fusion.^[^ ^9b,c^ ^]^ We first predicted the binding of the three domains of VCP, the N‐terminal domain, the D1 ATPase domain, and the D2 ATPase domain, to MFN1/2 on the PPA_Pred website. Among the three domains, the D2 ATPase domain containing the site K568 exhibited the strongest binding capacity to MFN1 and MFN2 (Table S1, Supporting Information). In addition, TNF‐*α* treatment attenuated the binding of VCP to MFN1, whereas the capacity of VCP to bind to MFN2 was not affected by TNF‐*α* (**Figure** **6**a,b). Both the cellular and mitochondrial levels of MFN1 were increased after TNF‐*α* treatment, whereas the treatment did not significantly change the levels of MFN2 (Figure 6c,d). VCP also reportedly promotes mitophagy by localizing mitochondria and recruiting LC3B.^[^ ^9a^ ^]^ However, the mitochondrial levels of VCP and the binding capacity of VCP to LC3B were not significantly changed in BMMSC after treatment with TNF‐*α* (Figure 6e,f). The binding of VCP to MFN1 was attenuated by VCP‐K658E but enhanced by VCP‐K658R compared with VCP‐Wt (Figure 6g). The cellular and mitochondrial levels of MFN1 were upregulated in BMMSC overexpressing VCP‐K658E, whereas the opposite results were obtained in BMMSC overexpressing VCP‐K658R (Figure 6h). These results suggest that the succinylation at K658 negatively affects the binding capacity of VCP to MFN1 and thus regulates its function in mitophagy.

### 2.7 The TNF‐*α*‐Induced Expression of KAT2A Regulates VCP Succinylation

**Figure 7:**
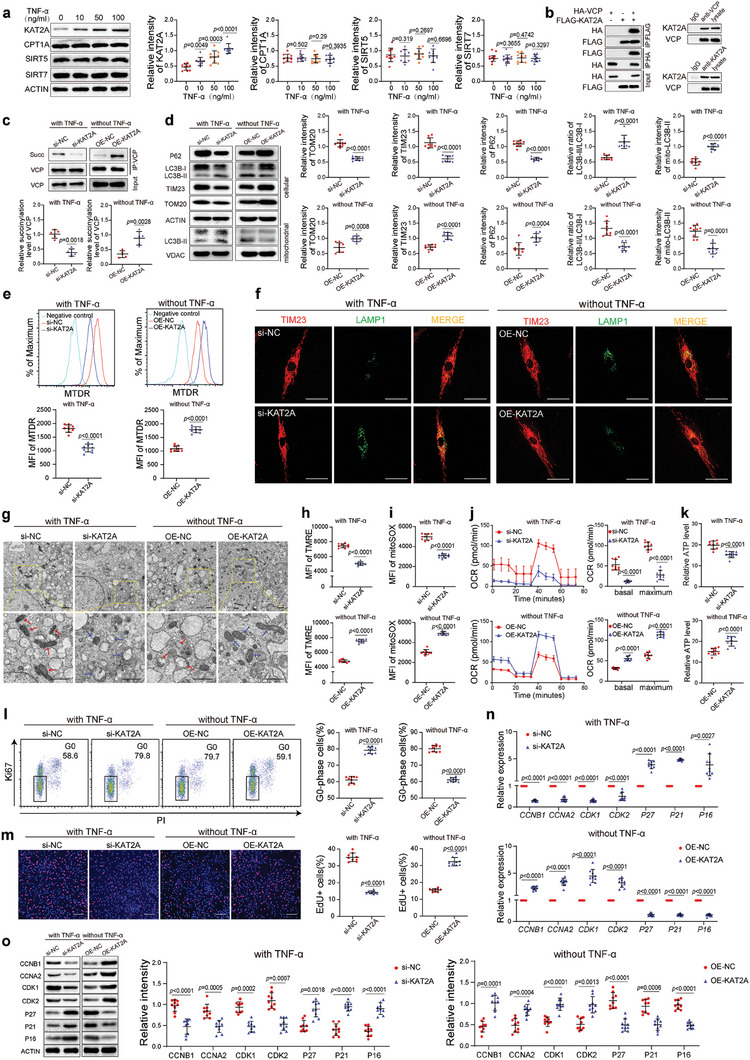
The TNF‐*α*‐induced expression of KAT2A regulates VCP succinylation. a) TNF‐*α* upregulated the protein level of KAT2A in hBMMSC in a concentration‐dependent manner, whereas the protein levels of CPT1A, SIRT5, and SIRT7 were not altered by TNF‐*α* (*n* = 9). b) Coimmunoprecipitation showed the interaction of VCP and KAT2A in HEK 293T cells (left) and hBMMSC (right). c) In the presence of TNF‐*α*, the knockdown of KAT2A (KD/with TNF‐*α*) downregulated the succinylation level of VCP, and in the absence of TNF‐*α* the overexpression of KAT2A (OE/without TNF‐*α*) upregulated the succinylation level of VCP (*n* = 5). d) KD/with TNF‐*α* decreased the protein levels of TOM20, TIM23, and P62 and increased the ratio of LC3B‐II/LC3B‐I and the level of mitochondria‐related LC3B‐II, while OE/without TNF‐*α* exhibited the opposite effect (*n* = 9). e) KD/with TNF‐*α* and OE/without TNF‐*α* reduced and elevated the MFI of MTDR in hBMMSC (*n* = 9), respectively. f) KD/with TNF‐*α* and OE/without TNF‐*α* promoted and inhibited the colocalization of mitochondria and lysosomes, respectively. Scale bar = 50 µm. g) KD/with TNF‐*α* and OE/without TNF‐*α* increased and decreased the number of fragmented mitochondria and mitochondria engulfed by autophagosomes, respectively. Scale bar = 2 µm. h–k) KD/with TNF‐*α* reduced the TMRE MFI (h), MitoSOX MFI (i), and the OCR (j), and elevated the ATP level (k) in hBMMSC (*n* = 9). OE/without TNF‐*α* exhibited the opposite effects. l, m) KD/with TNF‐*α* increased the proportion of G0‐phase hBMMSC (*n* = 9) and decreased the proportion of EdU+ hBMMSC (*n* = 9), while OE/without TNF‐*α* exhibited the opposite effects. Scale bar = 200 µm. n, o) KD/with TNF‐*α* and OE/without TNF‐*α* had opposite effects on the mRNA (n) and protein (o) levels of cell cycle‐related genes in hBMMSC (*n* = 9). The values are presented as the mean ± SD. The statistical analyses were performed as follows: one‐way ANOVA followed by Bonferroni's post hoc comparisons (a) and two‐tailed paired t‐test (c–e, h–o).

The main enzymes regulating protein succinylation reported to date are KAT2A, CPT1A, SIRT5, and SIRT7.^[^ ^10^, ^16^ ^]^ The expression of KAT2A was increased after treatment with TNF‐*α*, whereas there was no significant difference in the expression levels of CPT1A, SIRT5, or SIRT7 (**Figure** **7**a). To clarify whether KAT2A can regulate the succinylation level of VCP, the interaction of KAT2A with VCP was first verified by coimmunoprecipitation in HEK 293T cells overexpressing HA‐VCP and FLAG‐KAT2A. HA‐VCP could pull down FLAG‐KAT2A, and HA‐VCP was reciprocally pulled down by FLAG‐KAT2A (Figure 7b). The interaction of endogenous KAT2A and VCP was further verified in BMMSC (Figure 7b). The knockdown of KAT2A in the presence of TNF‐*α* decreased the succinylation level of VCP, whereas the overexpression of KAT2A in the absence of TNF‐*α* increased the succinylation level of VCP (Figure 7c). However, KAT2A had no regulatory effect on the level of VCP succinylation in BMMSC overexpressing VCP‐K658R (Figure S6a, Supporting Information). These results suggest that KAT2A elevates the succinylation level of VCP at K658.

Next, the effects of KAT2A on mitophagy were measured. The knockdown of KAT2A decreased the protein levels of TOM20 and TIM23 and the MFI of MTDR in the presence of TNF‐*α* (Figure 7d,e). Downregulated P62 levels, elevated LC3B‐II/LC3B‐I ratio, and mitochondria‐associated LC3B‐II levels were observed after the knockdown of KAT2A (Figure 7d). The colocalization of mitochondria with lysosomes and the number of fragmented and autophagocytosed mitochondria were increased in BMMSC with KAT2A knockdown (Figure 7f,g). The knockdown of KAT2A in the presence of TNF‐*α* also decreased the MFI of TMRE and MitoSOX, reduced the basal and maximum OCR, and increased the ATP level (Figure 7h–k). However, in the absence of TNF‐*α*, the overexpression of KAT2A showed effects opposite to those induced by KAT2A knockdown (Figure 7d–k). In addition, in BMMSC overexpressing VCP‐K658R, the knockdown and the overexpression of KAT2A failed to regulate mitophagy (Figure S6b–d, Supporting Information). These results suggest that KAT2A inhibits mitophagy by elevating the succinylation level of VCP at K658.

Furthermore, the KAT2A knockdown in the presence of TNF‐*α* significantly increased the proportion of G0‐phase BMMSC and decreased the proportion of EdU+ BMMSC (Figure 7l,m). The knockdown of KAT2A decreased the expression of CCNB1, CCNA2, CDK1, and CDK2, and increased the expression of P16, P21, and P27 (Figure 7n,o). The opposite effects were observed upon the overexpression of KAT2A in the absence of TNF‐*α* (Figure 7m–o). However, these regulatory effects of KAT2A were lost in BMMSC overexpressing VCP‐K658R (Figure S6e,f, Supporting Information). These results suggest that KAT2A impedes BMMSC quiescence by elevating the succinylation level of VCP at K658.

### 2.8 KAT2A Regulates BMMSC Quiescence and Function In Vivo

**Figure 8:**
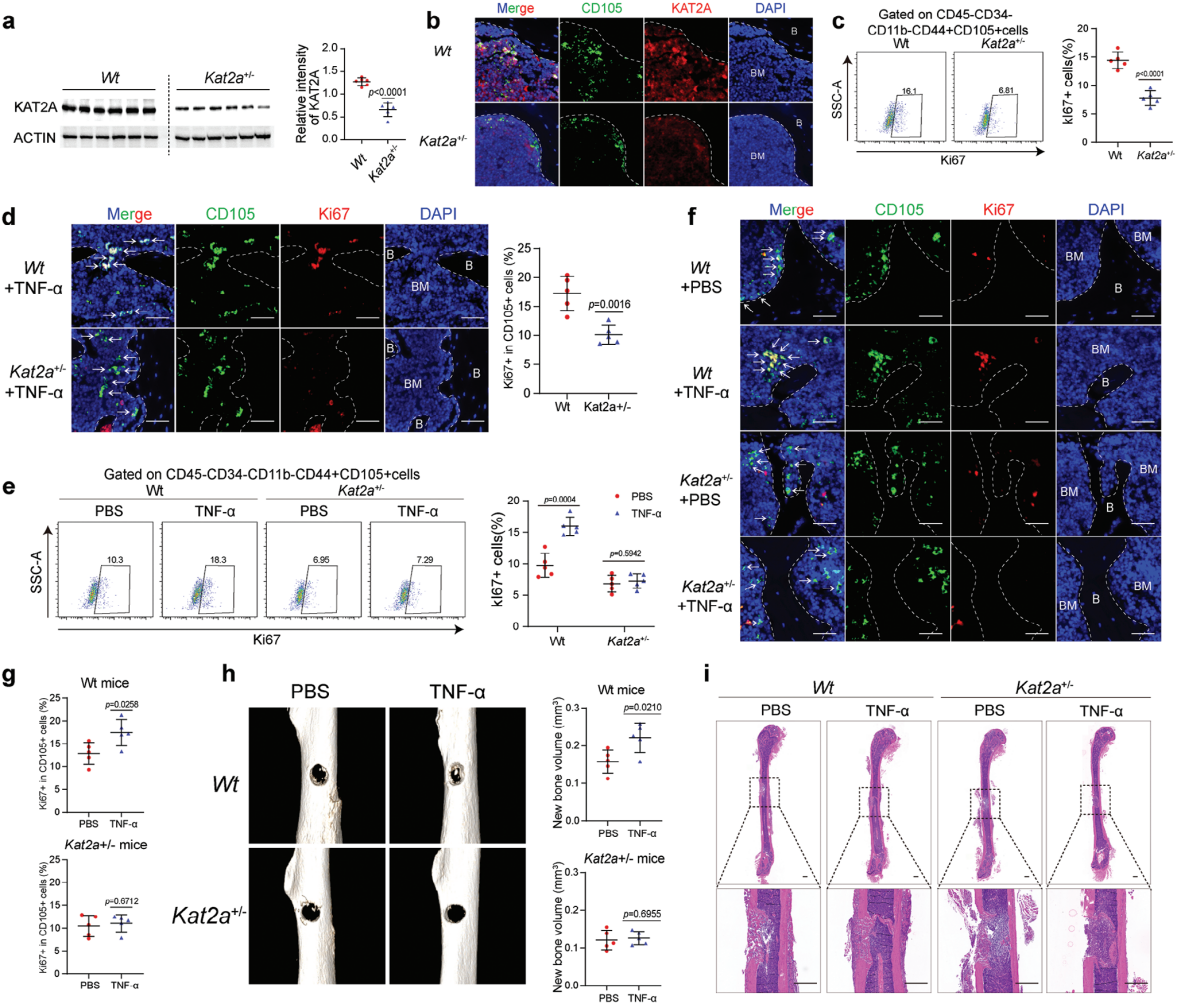
KAT2A regulates BMMSC quiescence and functional performance in vivo. a, b) Western blotting (a) and immunofluorescence (b) showed the decreased expression of KAT2A in mouse BMMSC (mBMMSC) from *Kat2a* ^+/−^ mice compared with those from wild‐type mice (*n* = 6). The dotted line represents the demarcation between the bone marrow and trabeculae, B indicates bone and BM indicates bone marrow. Scale bar = 50 µm. c, d) Flow cytometry analysis (c) and immunofluorescence assays (d) showed that the proportion of Ki67+ BMMSC (*n* = 5) was higher in wild‐type mice than in *Kat2a* ^+/−^ mice upon TNF‐*α* treatment. The dotted line represents the demarcation between the bone marrow and trabeculae, the arrows indicate BMMSC, B indicates bone and BM indicates bone marrow. Scale bar = 50 µm. e–g) Flow cytometry analysis (e) and immunofluorescence assays (f, g) showed that TNF‐*α* increased the proportion of Ki67+ BMMSC in wild‐type fracture mice and this effect was lost in *Kat2a* ^+/−^ fracture mice (*n* = 5). The dotted line represents the demarcation between bone marrow and trabeculae, the arrows indicate BMMSC, B indicates bone and BM indicates bone marrow. Scale bar = 50 µm. h, i) Micro‐CT (h) and H&E staining (i) showed that TNF‐*α* promoted fracture repair in wild‐type mice, and this effect was lost in *Kat2a* ^+/−^ mice (*n* = 5). Scale bar = 500 µm. The values are presented as the mean ± SD. The statistical analyses were performed with a two‐tailed Student's *t*‐test.

To explore the role of KAT2A in regulating BMMSC quiescence in vivo, *Kat2a* ^+/−^ mice were constructed. Decreased expression of KAT2A was observed in BMMSC from *Kat2a* ^+/−^ mice compared with BMMSC from wild‐type mice (**Figure** **8**a,b). In wild‐type mice, the local injection of 5 µg of TNF‐*α* into the femur for three consecutive days increased the proportion of Ki67+ BMMSC, but this effect was blocked in *Kat2a* ^+/−^ mice (Figures 1g,h and 8c,d). Previous studies revealed that the quiescent state of MSCs could affect their functions in vivo.^[^ ^1^, ^3^ ^]^ To explore whether quiescence affects the involvement of BMMSC in tissue repair in vivo, the femur fracture models were established in wild‐type and *Kat2a* ^+/−^ mice and 5 µg of TNF‐*α* or PBS were then locally injected into the fracture site for three consecutive days. The proportion of Ki67+ BMMSC in vivo was increased in the wild‐type mice injected with TNF‐*α* compared with those injected with PBS, whereas no difference in the proportion of Ki67+ BMMSC was found between *Kat2a* ^+/−^ mice treated with TNF‐*α* or PBS (Figure 8e–g). Two weeks after fracture, Micro‐CT was performed, and the results showed that the fracture defect area was markedly smaller in wild‐type mice injected with TNF‐*α* than in those injected with PBS (Figure 8h), as was also confirmed by H&E staining (Figure 8i). However, this therapeutic effect of TNF‐*α* on fracture was blocked in the *Kat2a* ^+/−^ mice (Figure 8h,i). These results suggest that KAT2A is involved in TNF‐*α*‐regulated BMMSC quiescence and function in vivo.

### 2.9 KAT2A Overexpression‐Activated BMMSC Exhibit More Prominent Therapeutic Functions than Quiescent BMMSC

**Figure 9:**
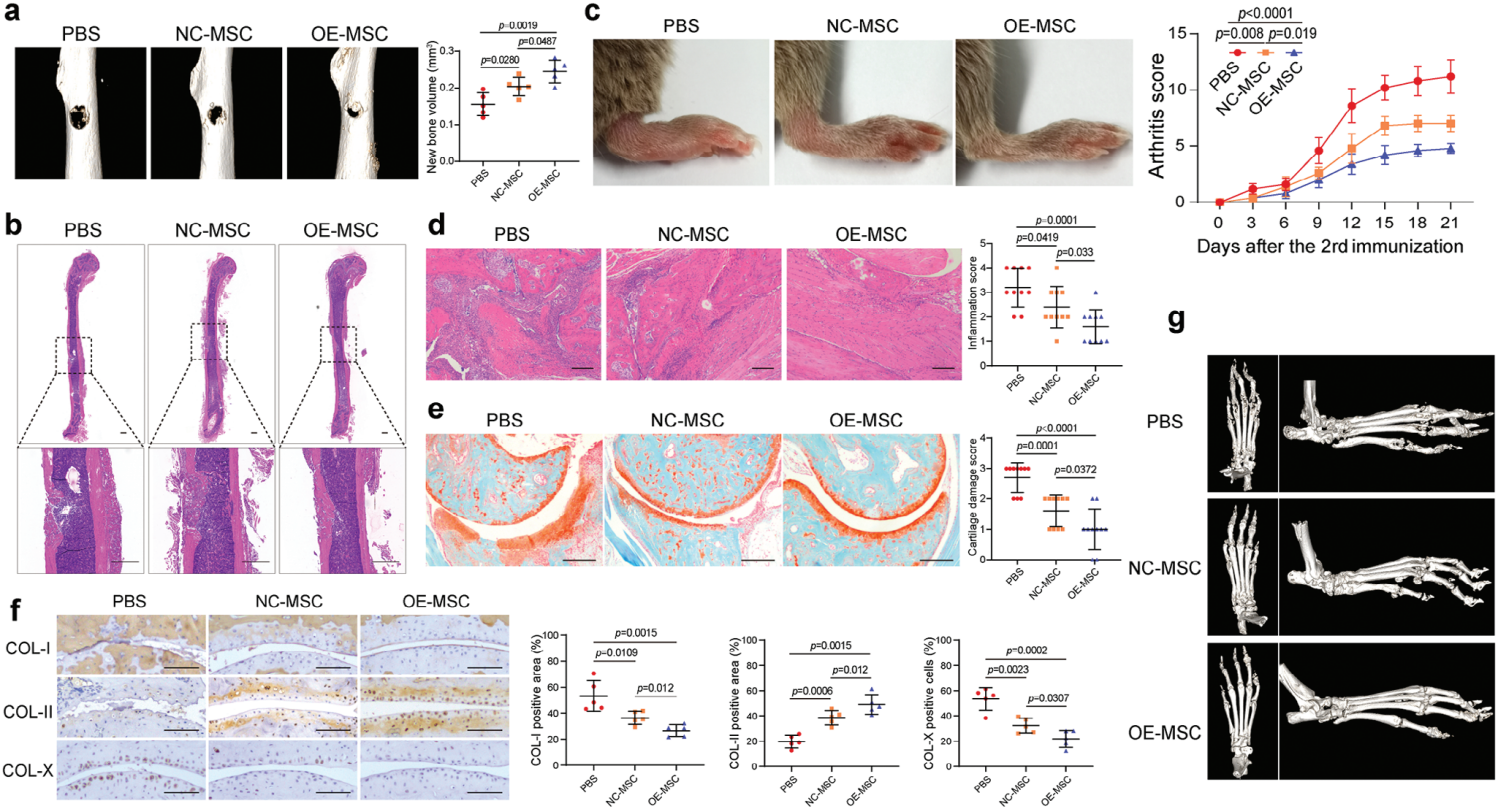
KAT2A overexpression‐activated BMMSC exhibit a more prominent therapeutic function than quiescent BMMSC a, b) Micro‐CT (a) and H&E staining (b) showed that mouse OE‐MSC exhibited a stronger fracture repair function than NC‐MSC (*n* = 5). Scale bar = 500 µm. c) Mouse OE‐MSC reduced the joint swelling and arthritis scores to a greater degree than NC‐MSC (*n* = 5). d) HE staining showed that mouse OE‐MSC more strongly attenuated inflammatory cell infiltration than NC‐MSC (*n* = 10). Scale bar = 200 µm. e) Safranin O‐fast green staining showed mouse OE‐MSC more prominently reduced cartilage degradation than NC‐MSC (*n* = 10). Scale bar = 200 µm. f) Immunohistochemical staining showed mouse OE‐MSC decreased the Collagen‐I positive area and Collagen‐X positive cells, and increased Collagen‐II positive area to a more pronounced extent than NC‐MSC (*n* = 5). Scale bar = 200 µm. g) Representative images of Micro‐CT showed that mouse OE‐MSC more strongly attenuated the destruction of joints than NC‐MSC. The values are presented as the mean ± SD. The statistical analyses were performed as follows: one‐way ANOVA followed by Bonferroni's post hoc comparisons (a, d–f) and Two‐way repeated‐measures ANOVA (c).

To evaluate the bone repair function of KAT2A overexpression‐activated BMMSC, osteogenesis capacity was first detected. Activation by TNF‐*α* enhanced ARS and alkaline phosphatase (ALP) staining, and these effects were blocked in BMMSC with KAT2A knockdown, in which the activation by TNF‐*α* was blocked (Figure S7a,b, Supporting Information). However, in the absence of TNF‐*α*, the activation of mouse BMMSC by KAT2A overexpression (OE‐MSC) enhanced ARS and ALP staining compared with quiescent mouse BMMSC with control vector overexpression (NC‐MSC) (Figure S7c, Supporting Information). Furthermore, the osteogenesis capacity of NC‐MSC and OE‐MSC were also measured in vivo. H&E, Masson, and Collagen‐I staining showed that OE‐MSC induced a larger osteogenesis area than NC‐MSC (Figure S7d, Supporting Information). These results suggest that the activation by TNF‐*α* or KAT2A overexpression enhances the osteogenic differentiation capacity of BMMSC. A bone fracture model was then constructed, and GFP‐labeled NC‐MSC or OE‐MSC were transplanted into the bone fracture site. NC‐MSC moderately reduced the bone defect area compared with PBS treatment alone, and this effect was further amplified by OE‐MSC (**Figure** **9**a,b). Furthermore, the involvement of the transplanted BMMSC in bone formation was detected. A higher expression level of OCN was observed in OE‐MSC than in NC‐MSC (Figure S7e, Supporting Information). These results suggest that KAT2A overexpression‐activated BMMSC exhibits a more prominent bone repair function than quiescent BMMSC.

To explore the therapeutic function of KAT2A overexpression‐activated BMMSC in autoimmune arthritis, their immunosuppressive properties were detected. BMMSCs were cocultured with peripheral blood mononuclear cells (PBMCs) stained with CFSE in the presence of anti‐CD3/CD28 microbeads and IL‐2. As revealed by the proliferation rate, activation by TNF‐*α* significantly enhanced the suppressive effects compared with those of quiescent BMMSC (Figure S7f, Supporting Information). However, in BMMSC with KAT2A knockdown, in which TNF‐*α* could not activate BMMSC, the enhanced suppressive effect of TNF‐*α*‐treated BMMSC on PBMC proliferation was blocked (Figure S7g, Supporting Information). Furthermore, OE‐MSC exhibited a significantly enhanced suppressive effect on PBMC proliferation compared with NC‐MSC (Figure S7h, Supporting Information). MSCs reportedly exert their immunoregulatory effects by restraining the differentiation of Th17, a cell type that plays an important role in autoimmune arthritis.^[^ ^17^ ^]^ Therefore, BMMSC were cocultured with naïve CD4+ T cells under Th17 polarization conditions and the differentiation of Th17 cells was detected. Quiescent BMMSC significantly inhibited Th17 differentiation, while this effect was more prominent in TNF‐*α*‐activated BMMSC (Figure S7i, Supporting Information). The enhanced inhibitory effect on Th17 differentiation was blocked in BMMSC with KAT2A knockdown (Figure S7j, Supporting Information). Furthermore, OE‐MSC exerted a stronger inhibitory effect on Th17 differentiation than NC‐MSC (Figure S7k, Supporting Information). These results suggest that activated BMMSC induced by TNF‐*α* and KAT2A overexpression exhibited enhanced suppressive effects on PBMC proliferation and Th17 differentiation compared with quiescent BMMSC. A collagen‐induced arthritis (CIA) model was then constructed, and NC‐MSC or OE‐MSC were transplanted through the tail vein. NC‐MSC reduced joint swelling, lowered arthritis scores, and reduced inflammatory cell infiltration compared with PBS, whereas OE‐MSC exerted stronger effects than NC‐MSC (Figure 9c,d). In addition, NC‐MSC alleviated cartilage degradation and bone destruction, and more significant effects were observed in the OE‐MSC group, as revealed by safranin red‐fast green staining, type I, II, and X collagen staining and Micro‐CT (Figure 9e–g). Furthermore, the in vivo immunosuppressive effects of BMMSC were explored. Splenocytes from CIA mice were extracted, and the Th17 frequency was detected. NC‐MSC significantly decreased the frequency of Th17 in CD4+ cells, and this effect was further amplified by OE‐MSC (Figure S7l, Supporting Information). These results suggest that KAT2A overexpression‐activated BMMSC exerts stronger effects on alleviating autoimmune arthritis than quiescent BMMSC.

## 3 Discussion

BMMSCs are pluripotent stem cells with immunoregulatory and multilineage differentiation functions and are widely used as seed cells for tissue repair and as therapeutic cells for inflammatory disease.^[^ ^1^, ^18^ ^]^ However, the functional capacity of stem cells relies on their ability to shift from a quiescent state to an activated state.^[^ ^19^ ^]^ In vivo, stem cells mainly remain in quiescence, a reversible cellular state of arrest in the G0 phase and functional dormancy.^[^ ^20^ ^]^ In response to external stimulation by cytokines, stem cells become activated and perform their powerful functions. As previously reported, TNF‐*α*, along with IFN‐γ and TGF‐β, can regulate

the quiescence of neural stem cells (NSC) and HSC.^[^ ^21^ ^]^ In our study, we further demonstrated that TNF‐*α* impeded the quiescence and promoted the activation of BMMSC, and this effect was enhanced with increases in the TNF‐*α* concentration and stimulation time. The functions of BMMSC are under the control of various cytokines, including TNF‐*α*, but TNF‐*α* may exert diverse effects on BMMSC function under different stimulation conditions.^[^ ^4^ ^]^ In any case, our results indicated that upstream TNF‐*α* first promotes rather than inhibits BMMSC activation, emphasizing both the triggering and regulatory roles of TNF‐*α* in BMMSC.

Stem cells undergo a shift in energy metabolism from quiescence to activation, and activated stem cells exhibited a higher energy demand compared with quiescent cells.^[^ ^5^ ^]^ The energy supply of quiescent BMMSC was previously reported to be predominantly glycolytic.^[^ ^22^ ^]^ During the activation of BMMSC induced by TNF‐*α*, the increased number of mitochondria and the enhanced activity of OXPHOS were positively correlated with the activation level of BMMSC, whereas inconsistent alterations were observed between the glycolysis levels and BMMSC activation, indicating that OXPHOS rather than glycolysis plays a more prominent role in energy metabolism in activated BMMSC, as was also observed during the activation of HSC and NSC.^[^ ^1^, ^8^ ^]^ BMMSC, along with HSC and NSC, exist in a hypoxic environment, and anaerobic glycolysis is suitable to meet the low energy requirements of quiescent BMMSC, HSC, and NSC.^[^ ^24^ ^]^ However, once activated, these stem cells perform many cellular activities.^[^ ^5^ ^]^ Although OXPHOS supplies energy at a lower rate than glycolysis, it is more productive and involves a greater tricarboxylic acid cycle influx, which can provide essential metabolites for proliferative activities.^[^ ^25^ ^]^ Thus, enhanced OXPHOS may be a more suitable energy metabolism pattern for these activated stem cells. However, satellite cells and lymphocytes, which are located in sites with a greater oxygen supply, rely mainly on OXPHOS for energy supplementation during quiescence.^[^ ^26^ ^]^ Simultaneous enhancement of glycolysis and OXPHOS was detected during the activation of satellite cells and lymphocytes, which undergo rapid proliferation, and this process requires both a rapid energy supply and large amounts of essential metabolites.^[^ ^26^, ^27^ ^]^ Therefore, the energy metabolism pattern of quiescent or activated stem cells is closely related to the environment in which the stem cells are located and the cellular activities that the stem cells undergo after activation. The enhanced OXPHOS activity in activated BMMSC is attributable to the inhibition of mitophagy and the increased number of mitochondria induced by TNF‐*α*. The inhibition of mitophagy is often accompanied by the accumulation of damaged mitochondria and impaired metabolic function, leading to cellular damage.^[^ ^28^ ^]^ However, a previous study showed that HSC maintain quiescence by removing healthy mitochondria to reduce metabolic levels, suggesting that quiescent stem cells may manage metabolism by actively removing healthy mitochondria rather than by passively removing damaged mitochondria.^[^ ^8c^ ^]^ In quiescent BMMSC, mitochondria may be healthy but subjected to high levels of autophagy, and after TNF‐*α* inhibits mitophagy, the number of healthy mitochondria increases, which results in the promotion of OXPHOS and the activation of BMMSC.^[^ ^22^ ^]^

How TNF‐*α* inhibits mitophagy is an important issue of concern. Cytokines can regulate protein succinylation,^[^ ^29^ ^]^ which reportedly regulates mitophagy and the cell cycle.^[^ ^11^, ^30^ ^]^ Our study showed that TNF‐*α* inhibited mitophagy and promoted OXPHOS by increasing the protein succinylation levels in BMMSC. However, the high protein succinylation levels induced by SIRT5 knockdown in precursor adipocytes reportedly promote mitophagy and inhibit OXPHOS.^[^ ^12^ ^]^ This is inconsistent with our results, probably because the induction of high succinylation levels in different cellular backgrounds or by different approaches leads to different protein succinylation profiles, resulting in different regulatory effects. To further explore the regulatory mechanism of TNF‐*α*‐induced protein hypersuccinylation on mitophagy, the protein succinylation profile was analyzed. Treatment with TNF‐*α* increased the succinylation level of site K658 of VCP, a mitophagy‐related protein, which was further verified to inhibit mitophagy. Previous studies have shown that VCP promotes mitophagy by translocating MFN1 out of mitochondria and promoting mitochondrial division.^[^ ^9b^ ^]^ Furthermore, K658 is located in the D2 ATPase domain, which is mainly associated with protein translocation, suggesting that succinylation at K658 may inhibit mitophagy by affecting VCP translocase function.^[^ ^31^ ^]^ Our study revealed that the succinylation of VCP at K658 inhibited the binding of VCP to MFN1 and increased the MFN1 protein levels in mitochondria.

The main enzymes that have been found to regulate succinylation modifications to date are KAT2A, CPT1A, SIRT5, and SIRT7,^[^ ^10^, ^16^ ^]^ which also reportedly regulate mitochondrial metabolism^[^ ^32^ ^]^ and BMMSC function.^[^ ^12^, ^33^ ^]^ We found that TNF‐*α* treatment upregulated the expression of KAT2A, and did not significantly alter the expression of other enzymes. Previous studies showed that KAT2A regulates the cell cycle in pancreatic cancer cells through its succinylase function.^[^ ^34^ ^]^ In our study, knockdown of KAT2A in the presence of TNF‐*α* reduced the succinylation level of VCP at K658, and this effect promoted mitophagy, inhibited OXPHOS, and suppressed the activation of BMMSC by TNF‐*α*, whereas the overexpression of KAT2A in the absence of TNF‐*α* led to the opposite results, suggesting that KAT2A mediates the activation of BMMSC by TNF‐*α* through its succinylase function. The regulation of quiescence by KAT2A was also reported in HSC. However, in HSC, KAT2A promoted HSC quiescence by acetylating PGC‐1*α*, reducing mitochondrial mass, and decreasing OXPHOS activity, which are effects opposite to those found in our study, suggesting that the regulatory effect of KAT2A on mitochondrial metabolism and stem cell quiescence is dependent on the specific stem cell context and on its enzymatic catalytic function.^[^ ^14^ ^]^

As multipotent stem cells that possess multidirectional differentiation and immunoregulatory functions, BMMSCs are widely used in the clinic, for applications such as bone repair and the treatment of autoimmune diseases.^[^ ^1^, ^35^ ^]^ However, the effect of the quiescent versus activated states of BMMSC on therapeutic outcomes has not been emphasized. It is likely that these infused BMMSCs are primed by various cytokines in vivo once injected. The present study showed that BMMSC activated by KAT2A overexpression have stronger fracture repair and immunoregulatory functions than quiescent BMMSC. In addition, the knockdown of KAT2A in mice led to a decrease in BMMSC activation after TNF‐*α* injection, and to deficient bone repair ability. These results suggest that activation is essential for the therapeutic function of BMMSC. Thus, our study provides new insight showing that activation is key to improving the effectiveness of BMMSC in clinical applications and that KAT2A is an important target.

The limitations of this study should be acknowledged. First, TNF‐*α* is a crucial immune factor and TNF‐*α*‐related immune responses may be involved in the activation of BMMSC. Although TNF‐*α* can directly act on and activate BMMSC through KAT2A without the involvement of other immune cells and factors in vitro, the results from the in vivo experiments, which showed that the TNF‐*α*‐induced activation of BMMSC is blocked in *Kat2a* ^+/−^ mice, cannot exclude the involvement of other immune cells and factors in this process. Second, the blockade of therapeutic effects of TNF‐*α* on fracture in *Kat2a* ^+/−^ mice suggested the importance of the activation of BMMSC in TNF‐*α*‐promoted fracture repair to some extent. However, immune cells, including macrophages and T cells, are involved in fracture repair, and the development and functions of these immune cells are regulated by KAT2A.^[^ ^36^ ^]^ It was also possible that the blockade of the therapeutic effects of TNF‐*α* on fracture in *Kat2a* ^+/−^ mice can be attributed to aberrant immune responses caused by KAT2A deficiency. Further investigation was needed to address these limitations, and the construction of conditional knockout mice with specific depletion of KAT2A or TNFR1 in BMMSC may help to further ascertain the relationship among TNF‐*α*, KAT2A, and the bone repair function of BMMSC

## 4 Conclusion

In conclusion, our study found that TNF‐*α* increased KAT2A expression in BMMSC, which mediated the succinylation of VCP at K658 to inhibit mitophagy and promote OXPHOS. This mechanism results in impeding the quiescence and elevating the activation of BMMSC, which enhances the potential of BMMSC to contribute to bone repair and immunoregulation in vivo. Our study further elucidates the mechanisms regulating BMMSC quiescence and provides new insights and targets for improving the clinical application of BMMSC.

## 5 Experimental Section

### Study Approval

The animal experiment protocols were approved by the Institutional Animal Care and Use Committee of Sun Yat‐Sen University. The approval numbers of the animal experiments were as follows: 2022001363, 2022002390, 2023002659, and 2023002660. The collection of human specimens and the experiments with human specimens were approved by the Ethics Committee of the Eighth Affiliated Hospital, Sun Yat‐Sen University. The approval numbers for handling human subjects were 2021r037 and 2022r023.

### Mice

BALB/c nu/nu mice, DBA/1 mice, wild‐type C57BL/6 and *Kat2a* ^+/−^ C57BL/6 mice were purchased from GemPharmatech and were maintained in a specific pathogen‐free barrier facility for the experiments conducted in this study.

### Collagen‐Induced Arthritis (CIA) Model

For induction of the CIA model, two immunizations were administered to DBA/1 mice (male, 6–8 weeks, 20–30 g). The initial immunization was performed on the first day. Equal volumes of chicken type II collagen (Chondrex, #20012) and Freund's complete adjuvant (Chondrex, #7001) were added to a 15 mL‐centrifuge tube and vigorously stirred using a high‐speed homogenizer (VRera, #FSH‐2A) at 10 000 rpm for 2 min on ice followed by 1 min‐interval. The cycle of stirring and interval was repeated three times to ensure adequate emulsification of chicken type II collagen and Freund's complete adjuvant at low temperatures. Then, 100 µL of the homogenate of chicken type II collagen and Freund's complete adjuvant was injected subcutaneously into the tail at a site 2 cm from the root of the tail. On day 21, the second immunization was performed. Equal volumes of chicken type II collagen and Freund's incomplete adjuvant (Chondrex, #7002) were emulsified as described above, and 100 µL of homogenate was slowly injected subcutaneously into the tails while avoiding the dilated blood vessels caused by the initial immunization. The inflammation of each joint was scored every 3 days using the following scoring criteria: 0, normal; 1, redness or inflammation of one joint; 2, redness or inflammation of more than one joint; 3, redness or inflammation of the entire paw; and 4, ankylosis or deformity. The score for each mouse was the sum of the limb scores.

### Fracture Model

For the construction of the fracture model, the mice were anesthetized by intraperitoneal injection of 1% sodium pentobarbital at a dose of 50 mg kg^−1^. After disinfection with iodophor, the skin on the back of the thigh was cut, and the soft tissues posterior to the femur were bluntly separated. A 1 mm‐diameter hole was drilled in the middle of the femur using a bone drill at a slow speed, and during this process, saline was sprayed onto the surgical site to maintain the local tissues at normal temperature. Once the drill had just penetrated the cortical bone, the drilling process was stopped, and the site was then rinsed with iodophor. Afterward, PBS/TNF‐*α* (PrimeGene, #123‐01) or mouse BMMSC was administered to the fracture site as indicated. Then the soft tissues were anatomically reset, and the skin was sutured.

### Isolation, Culture, and Identification of Mouse BMMSC

The mice were subjected to cervical dislocation following anesthesia with isoflurane. The mice were then sterilized with 70% ethanol for 5 min. Mouse BMMSC were isolated from the femurs and tibias of wild‐type or KAT2A^+/−^ C57BL/6 mice (male, 6–8 weeks, 20–30 g). The femurs and tibias were dissected and cleaned of connective tissue. Both ends of the femurs and tibias were incised, and the bone marrow cells were washed out with PBS, and filtered through a 70‐µm cell strainer. The collected cells were centrifuged at 1500 rpm for 5 min, resuspended in a complete MesenCult expansion medium (STEMCELL, # 05513) containing 10% FBS, 100 IU mL^−1^ penicillin, and 100 IU mL^−1^ streptomycin, and seeded into 25 cm^2^ flasks and cultured at 37°C in an atmosphere with 5% CO_2_. The nonadherent cells were removed after 3 days, and half of the medium was refreshed every 3 days. At 90% confluence, the cells were passaged using 0.05% trypsin. The phenotypes of the expanded mouse BMMSC were detected by flow cytometry analysis. BMMSC were harvested, washed with PBS, and incubated with anti‐CD45‐PE (BioLegend, #103105), anti‐CD11b‐FITC (BioLegend, #101205), anti‐CD34‐PE (BioLegend, #119307), anti‐CD29‐FITC (BioLegend, #102205), anti‐CD105‐FITC (Invitrogen, #MA5‐17945), anti‐Sca1‐PE (BioLegend, #108107) and anti‐CD44‐PE (BioLegend, #103023), and analyzed by flow cytometry.

### Mouse BMMSC Transplantation

Passage 2 BMMSC was digested using 0.05% trypsin and collected by centrifugation at 1500 rpm for 5 min. The cells were reseeded into 25 cm^2^ flasks and transfected with a GFP‐labeled control vector‐ (NC‐MSC) or KAT2A‐overexpression lentiviruses (OE‐MSC). After 3 days of culture, the overexpression efficiency was validated, and BMMSCs were then harvested. For treatment of the fracture model, BMMSC were counted and resuspended in PBS at a concentration of 10^5^ cells µL^−1^. A total of 10^6^ BMMSC in a volume of 10 µL were injected into the fracture site during surgery. For treatment of the CIA model, BMMSC were counted and resuspended in PBS at a concentration of 10^4^ cells µL^−1^, and a total of 10^6^ BMMSC in a volume of 100 µL were injected into the mice through the tail vein at a low rate, after the second immunization.

### Isolation, Culture, and Identification of Human BMMSC

20 healthy bone marrow donors were informed of the possible risks in advance. Human bone marrow samples were obtained by bone marrow puncture, and heparin was added immediately for anticoagulation. The bone marrow was then mixed with an equal volume of saline. The mixture was added to a 6% hydroxyethyl starch solution mixed upside down and allowed to stand for 40 min. The clarified solution of the middle layer was aspirated and centrifuged at 2000 rpm for 20 min. The supernatant was discarded and the precipitated cells were washed with PBS and resuspended in StemPro BMMSC SFM XenoFree medium (Thermo Fisher, #A1067501) supplemented with 10% FBS, 100 IU mL^−1^ penicillin, and 100 IU mL^−1^ streptomycin. The cells were then seeded into 25 cm^2^ flasks and cultured at 37 °C in an atmosphere with 5% CO_2_. The nonadherent cells were removed after 5 days, and the medium was refreshed every 3 days thereafter. Cells at 90% confluence were digested using 0.05% trypsin and 0.02% EDTA and reseeded into 75‐cm^2^ flasks. Cell surface markers of the expanded BMMSC were detected by flow cytometry analysis. BMMSC was digested using 0.05% trypsin and washed with PBS. The cells were then incubated with anti‐CD45‐PE (eBioscience, #12‐0459‐42), anti‐CD14‐PE (eBioscience, #12‐0149‐42), anti‐HLA‐DR‐FITC (BioLegend, #980402), anti‐CD29‐FITC (eBioscience, #11‐0299‐42), anti‐CD105‐PE (eBioscience, #12‐1057‐42), and anti‐CD73‐PE (BioLegend, #344003) and analyzed by flow cytometry.

### Induction of BMMSC Quiescence and Activation

BMMSC were seeded in six‐well plates at a density of 1 × 10^5^ cells/well. After 3 days of serum deprivation to induce quiescence, the medium was refreshed, and 10% FBS was added. Simultaneously, TNF‐*α* (PeproTech, #300‐01A) and different experimental conditions were applied were applied for the activation of BMMSC. In the TNF‐*α* withdrawal experiment, TNF‐*α* was removed after treatment for 24 h, and the cells were subjected to serum deprivation for another 3 days. The BMMSC was then cultured with 10% FBS for another 24 h.

### RNA Preparation and Quantitative Real‐Time PCR

Total RNA was isolated from BMMSC using TRIzol reagent (Invitrogen, #15596‐026) and reverse transcribed using Evo M‐MLV RT Master Mix (AG, #11706) according to the manufacturer's protocols. RT‐qPCR was performed using the SYBR Green Premix Pro Taq HS qPCR Kit (AG, #11718). Quantification was performed using the 2^−△△^Ct method, and the data were normalized to the levels of *ACTIN*. The primer sequences for each gene are shown in Table S2 (Supporting Information).

### Western Blotting Analysis

Cells were harvested in RIPA lysis buffer (Sigma–Aldrich, # R0278) containing 1% protease inhibitors (CWBIO, #CW2200S) and 1% phosphatase inhibitors (CWBIO, #CW2383S) for 30 min at 4 °C. To measure the protein succinylation levels, 10 µm trichostatin A (MCE, #HY‐15144) and 50 µm nicotinamide (Sigma, #N3376) were added. The cell lysates were centrifuged at 11000×g for 30 min at 4 °C, the supernatant was collected and protein concentration was measured using a BCA Protein Assay Kit (CWBIO, #CW0014S) according to the manufacturer's protocol. The supernatant was then mixed with sample loading buffer (Beyotime, #P0015) and boiled. For immunoprecipitation, the supernatant of the cell lysates was precleaned with protein A/G beads (Invitrogen, #88802) and then incubated with the indicated antibodies or control IgG overnight at 4 °C, and after the addition of protein A/G beads and the mixture was incubated for 3 h. The immunoprecipitates were washed, mixed with sample loading buffer, and boiled. Equal amounts of protein were separated by electrophoresis on SDS‐PAGE polyacrylamide gels and transferred to PVDF membranes (Millipore, #IPVH00010). After blocking with 5% BSA solution, the membranes were incubated with primary antibodies overnight at 4 °C and then with horseradish peroxidase (HRP)‐conjugated secondary antibodies for 1 h. The antibody‐antigen complexes were visualized and detected using an ECL reagent (Millipore, #WBKLS0500). The following antibodies were used: anti‐CCNB1 (Abcam, #ab181593), anti‐CCNA2 (Abcam, #ab181591), anti‐CDK1 (Abcam, #ab1333270), anti‐CDK2 (Abcam, #ab32147), anti‐P16 (ABclonal,#A11651), anti‐P21 (Abcam, #ab109520), anti‐P27 (ABclonal, #A16633), anti‐TIM23 (ABclonal, #A8688), anti‐TOM20 (ABclonal, #A19403), anti‐P62 (ABclonal, #A19700), anti‐LC3B (ABclonal, #A19665), anti‐VDAC1 (ABclonal,#A19707), anti‐VCP (Abcam, #ab109240), anti‐succinyllysine (PTMBIO, #PTM‐401), anti‐HA (CST, #3724S), anti‐Flag (CST, #14793S), anti‐MFN1 (ABclonal, #A9880), anti‐MFN2 (ABclonal, #A19678), anti‐KAT2A (Abcam, #ab217876), anti‐CPT1A (Abcam, #ab220789), anti‐SIRT5 (Abcam, #ab259967), anti‐SIRT7 (Abcam, #ab259968), anti‐β‐ACTIN (Abcam, #ab8226), HRP‐conjugated goat anti‐rabbit IgG (BOSTER, #BA1054), HRP‐conjugated goat anti‐mouse IgG (BOSTER, #BA1050), and control rabbit IgG for immunoprecipitation (Beyotime, #A7016). For western blotting, the primary antibodies were used at 1:1000 dilution, and the secondary antibodies were used at 1:3000 dilution. For immunoprecipitation, the antibodies were used at 1:100 dilution.

### Flow Cytometry

For detection of the quiescence status of BMMSC in vitro, the cells were digested with 0.05% trypsin and 0.02% EDTA and then centrifuged at 1500 rpm for 5 min. The collected BMMSC were resuspended in ethanol at −20 °C and incubated at 4 °C for 1 h for fixation, and then incubated with permeabilization buffer (Invitrogen, #GAS002S5) for 30 min. The permeabilized cells were resuspended in PBS‐BSA buffer (PBS containing 1% BSA) and stained with 1% anti‐Ki67‐BV‐421 (eBioscience, #48‐5698‐82) for 30 min. The cells were then washed with PBS, resuspended in 200 µL of propidium iodide solution containing RNase A (PI, BD, #550825), and analyzed within 30 min using a BD FACSCelesta cytometer.

For analysis of the activation status of BMMSC in vivo, the bone marrow cells were extracted. The femurs were dissected and cleaned of connective tissue. Both ends of the femurs were incised, and the bone marrow cells were washed out with PBS, and filtered through a 70‐µm cell strainer. The cells were centrifuged at 1500 rpm for 5 min. The collected cells were incubated with 2 µg of anti‐CD34‐Biotin (BioLegend, #128604), 1 µg of anti‐CD45‐Biotin (BioLegend, #103103), and 0.5 µg of anti‐CD11b (BioLegend, #101203) in a total volume of 200 µL for 25 min at 4 °C. The cells were washed with PBS and resuspended in 180 µL of separation buffer containing 0.5% BSA and 2 mm EDTA. 20 µL of anti‐biotin microbeads (Miltenyi Biotec, #130‐090‐485) was added to the cells and incubated for 25 min at 4 °C. The magnetically labeled cells were then removed using a separation column (Miltenyi Biotec, #130‐042‐401) in a magnetic field, and the CD34‐CD45‐CD11b‐cells were collected. After 15 min of staining with live/dead 780 dye (Invitrogen, #L34992), the cells were incubated with 1 µg of anti‐CD44‐PE and 0.5 µg of anti‐CD105‐FITC in a total volume of 100 µL for 30 min at room temperature away from light. The cells were then fixed and permeabilized as described above and stained with anti‐Ki67‐BV421 for 30 min. The cells were washed with PBS, resuspended in 200 µL of PI, and analyzed within 30 min using a BD FACSCelesta cytometer.

For measurement of the mitochondrial mass, the mitochondrial membrane potential, and the mitochondrial ROS, the cells were incubated with 100 nm MitoTracker Deep Red (Invitrogen, # M22426), 100 nm TMRE (MCE, # HY‐D0985A) and 1 µm MitoSOX (Invitrogen, # M36008) for 30 min at 37 °C, respectively. The cells were washed twice with PBS‐BSA buffer and then analyzed using a BD FACSCelesta cytometer.

### EdU Assay

The EdU assay was performed with an EdU Cell Proliferation Kit (Beyotime, #C0075). The BMMSC were added with 5 µm of EdU and cultured in the indicated condition for 24 h. After fixation with 4% paraformaldehyde for 20 min and permeabilization with 0.5% Triton X‐100 for 25 min, the cells were incubated with 1 mL of Click Reaction solution (860 µL of Click Reaction buffer, 40 µL of CuSO4, 2 µL of Azide 555 and 100 µL of Click Additive Solution) for 30 min away from light. After three washes with PBS, the cells were stained with 0.1% Hoechst 33342 for 10 min. The cells were washed with PBS and cell images were captured with a fluorescence microscope (Leica‐DMi8).

### RNA Sequencing and Data Analysis

Total RNA was isolated from BMMSC as described above. The PolyA+ RNA fraction was processed, and the cDNA library was constructed, and sequenced by the Beijing Genomics Institute using the BGISEQ500 platform. Low‐quality reads, splice contamination, and reads with a high unknown base N content were removed with SOAPnuke (v1.5.2). The clean reads were mapped to GCF_000001405.38_GRCh38.p12 using HISAT (v2.0.4) and aligned to the reference coding gene set using Bowtie (v2.2.5). Differentially expressed genes were analyzed using DESeq2 (v1.4.5) based on a fold change cutoff of 2 and an adjusted P value cutoff of 0.05. Clustering analysis, principal component analysis, gene ontology, and gene set enrichment analyses (GSEA) were performed using Dr. Tom (BGI).

### ATP Measurement Assay

The ATP levels in cells were measured using an ATP Assay Kit (Beyotime, #S0026). In brief, the cells were lysed and centrifuged at 12000×g for 5 min at 4 °C. Then, 20 µL of the supernatant was added to 180 µL of ATP detection working buffer and the mixture was transferred to each well of a 96‐well plate, followed by detection with a luminometer (BIOTEK Synergy HTX). The ATP levels were indicated as relative light units and were normalized to the protein concentrations.

### Oxygen Consumption Rate (OCR) and Extracellular Acidification Rate (ECAR) Measurement

The Seahorse XF Cell Mito Stress Test Kit (Seahorse, #103015‐100) and Seahorse XF Glycolysis Stress Test Kit were used for OCR and ECAR measurements, respectively. BMMSC (1 × 10^4^) were plated in each well of an XF96 plate. After treatment according to the experimental objective, BMMSC were loaded into the Seahorse XFe96 analyzer (Agilent) for OCR and ECAR measurement. The cells were subjected to Mito stress through the sequential addition of 1 µm oligomycin, 1 µm carbonyl cyanide p‐trifluoromethoxyphenylhydrazone (FCCP), and 150 nm rotenone. The oxygen concentration was measured after each addition, and the data were normalized to the protein concentration in each well. The basal OCR was calculated as the difference between the OCR measured before the addition of oligomycin and that measured after the addition of rotenone, and the maximum OCR was calculated as the difference between the OCR measured after the addition of FCCP and that measured after the addition of rotenone. For measurement of the ECAR, the cells were sequentially treated with 10 mm glucose, 1 µm oligomycin, and 50 mm 2‐deoxy‐glucose (2‐DG). The ECAR was detected after each addition, and the data were normalized to the protein concentration in each well. The basal ECAR was calculated as the difference between the ECAR measured before and after the addition of glucose. The maximum ECAR was calculated as the ECAR measured before the addition of glucose and that after the addition of oligomycin.

### Measurement of Lactate Production

Lactate production was measured using a Lactic Acid Assay Kit (Abbkine, #ktb1100) according to the manufacturer's protocols. Briefly, 50 µL of the cell supernatant was added to a mixture of 50 µL Lactate Assay Buffer and 50 µL of Working Reagent in a 96‐well plate and incubated at 37 °C for 30 min away from light. The absorbance of each well at 450 nm was measured using a luminometer (BIOTEK Synergy HTX). The production of lactate was normalized to the protein concentration.

### Measurement of Glucose Uptake

BMMSC cultured under the indicated conditions were starved using a glucose‐free medium (GIBCO, #11966025) for 1 h and then incubated with 2‐N‐(7‐nitrobenzo‐2‐oxa‐1,3‐diazole‐4‐amino)‐2‐deoxyglucose (2‐NBDG) at a final concentration of 100 µm at 37 °C for 30 min away from light. The cells were then digested with 0.05% trypsin and centrifugated at 1500 rpm for 5 min. The collected cells were analyzed by flow cytometry using a BD FACSCelesta cytometer.

### Cell Immunofluorescence Staining

For cell immunofluorescence staining, BMMSC in confocal dishes were fixed with 4% paraformaldehyde and permeabilized with 0.5% Triton X‐100. The cells were blocked with 5% BSA solution and then incubated with primary antibodies overnight at 4 °C. The cells were incubated with fluorescein‐labeled secondary antibodies for 1 h at room temperature away from light. Images were captured using a confocal imaging system (Nikon Eclipse Ni‐E). The following primary antibodies were used at the concentrations indicated in the instruction for each antibody: anti‐TOM20 (ABclonal, #A19403) anti‐LAMP1 (Abcam, #ab25630). The following secondary antibodies were used at 1:500 dilution: Alexa Fluor 488‐conjugated goat anti‐rabbit IgG secondary antibody (Invitrogen, #A‐11008) and Alexa Fluor555‐conjugated goat anti‐mouse IgG secondary antibody (Invitrogen, #A‐21422).

### Electron Microscopy

BMMSC in six‐well plates were harvested, centrifuged at 300×g for 5 min, and fixed in electron microscopy fixative solution (Thermo Fisher, #ED‐8484) for 30 min at room temperature. The cells were then postfixed with osmium tetroxide and dehydrated through a gradient of ethanol concentrations. The cells were subsequently embedded in epoxy resin, sectioned, and counterstained with 3% uranyl acetate‐lead citrate. Images were obtained using a transmission electron microscope system (Hitachi).

### RNA Interference

To silence the target genes, targeting and nontargeting siRNAs were synthesized by GenePharma. For gene silencing in 1 × 10^5^ BMMSC, 0.2 nmol siRNA and 4 µL of Lipofectamine RNAiMAX (Invitrogen, #13778150) were incubated in 100 µL of Opti‐MEM Reduced Serum Medium (Thermo Fisher, #31985070) for 5 min at 37 °C separately. The siRNA and Lipofectamine RNAiMAX were then mixed, incubated for 20 min at 37 °C, and then added to the cells. The cells were cultured in Opti‐MEM Reduced Serum Medium for 6 h and then switched to DMED containing 10% FBS. Three days after transfection, the knockdown efficiency was measured, and the cells were used in subsequent experiments. Nontargeting siRNA was used as a control. The siRNA sequences used in this study were as follows: VCP, 5′‐GCGGAGAGUGAAUUACCAATT‐3′; KAT2A, 5′‐GCUACCUACAAGGUCAAUUTT‐3′; and control nontargeting siRNA, 5′‐UUCUCCGAACGUGUCACGUTT‐3′.

### Lentivirus Construction and Transfection

VCP‐Wt, VCP‐K18E, VCP‐K18R, VCP‐K658E, VCP‐K658R, human KAT2A and mouse KAT2A overexpression lentiviruses and their vector controls were constructed by OBiO Technology. The lentivirus and vector control were added to DMEM with 5 µg mL^−1^ polybrene (OBiO, #HYFW2022101201) at a multiplicity of infection of 30 and incubated with the BMMSC for 24 h. The medium was refreshed on the second day. Three days after transfection, the transfection efficiency was analyzed, and subsequent experiments were performed.

### Mass Spectrometry Data Analysis

BMMSC were harvested, and after the addition of 8 m urea containing 1% protease inhibitors, 1% phosphatase inhibitors, 3 µm trichostatin A, and 50 mm nicotinamide, the cells were lysed by sonication at 4 °C. The samples were centrifuged at 12000×g for 10 min at 4 °C, and the supernatant was collected. The protein concentration was determined using a BSA kit (CWBIO, #CW0014s). For precipitation, the proteins were incubated with 20% TCA for 2 h at 4 °C and washed with acetone. The precipitates were air‐dried, and 200 mm TEAB was added. Trypsin was added at a ratio of 1:50 (protease: protein, m/m) for enzymolysis overnight at 4 °C. The peptides were reduced by incubation with 5 mm dithiothreitol for 30 min at 56 °C. Iodoacetamide was added to a concentration of 11 mm and the samples were incubated for 15 min at room temperature away from light. The peptides were desalted with a Strata X C18 SPE column (Agela‐Phenomenex, #00B‐S001‐A0) and vacuum freeze‐dried. The peptides were then dissolved, separated using an ultrahigh‐performance liquid system (NanoElute), and injected into an NSI source for ionization, and liquid chromatography–mass spectrometry (LC/MS) was performed to scan the peptides. The electrospray voltage was set as 1.75 kV, and the scanning range of the secondary mass spectrometry was set as 400 to 1500 m/z. The parallel cumulative serial fragmentation (PASEF) mode was used for data acquisition. One primary MS acquisition was followed by 10 PASEF mode secondary MS acquisitions with 30 s dynamic exclusion. The identification and quantification of proteins and PTM sites were performed using the MaxQuant search engine (v1.5.2.8). The allowable number of missed cut sites was set as 2. The peptide length cutoff was set as 7 amino acid residues, and the maximum number of peptide modifications was set as 5. Mass error tolerance for the primary parent ion and the main search were set as 20 and 5 ppm, respectively. The mass error tolerance of the secondary fragment ion was set as 0.02 Da. The identifications were filtered to 1% FDR. The data were analyzed using InterProScan, KEGG Mapper, WoLF PSORT, CELLO, and the R pPackage pheatmap.

### Induction of BMMSC Differentiation

For osteogenic induction, BMMSC were seeded in 12‐well plates at a density of 5 × 10^4^ cells/well and cultured in DMEM containing 10% FBS, 100 IU mL^−1^ penicillin, 100 IU mL^−1^ streptomycin, 0.1 µm dexamethasone (Selleck, #S1322), 10 mm *β*‐glycerol phosphate (Sigma, #G9422) and 50 µm ascorbic acid (Sigma, #A0278). The osteogenic induction medium was refreshed every 3 days.

For adipogenic induction, BMMSC were seeded in 12‐well plates at a density of 6×10^4^ cells/well and cultured in DMEM containing 10% FBS, 100 IU mL^−1^ penicillin, 100 IU mL^−1^ streptomycin, 1 µm dexamethasone, 10 µg mL^−1^ insulin (BI, #41‐975‐100), 0.2 mm indomethacin (APExBIO, #A8449) and 0.5 mm IBMX (APExBIO, #B7206). The adipogenic induction medium was refreshed every 3 days.

For chondrogenic induction, BMMSC were dotted on the center of each well of 24‐well plates at a density of 1 × 10^5^ cells/well in a volume of 10 µL. The BMMSC were allowed to adhere to the wells for 2 h, and DMEM containing 10% FBS, 100 IU mL^−1^ penicillin, 100 IU mL^−1^ streptomycin, 1% Insulin Transferrin Selenium‐A (ITS; Invitrogen, # 51300044), 50 mg mL^−1^ ascorbic acid and 10 ng mL^−1^ TGF‐β3 (PeproTech, #100‐36E) was added. The chondrogenic induction medium was refreshed every 3 days.

### Alizarin Red S (ARS) Staining and Quantification

After 14 days of osteogenic induction, the original medium was discarded and cells were washed three times with PBS. The cells were then fixed with 4% paraformaldehyde for 30 min and stained with Alizarin Red Staining Solution (Solarbio, # G8550) for 15 min. The staining solution was discarded, and the cells were washed three times with PBS and images were captured using a microscope. For ARS quantification, the cells were added with 10% Cetylpyridinium chloride solution (CPC, Sangon Biotech, #A600106) and shaken rapidly for 1 h at room temperature for adequate extraction of ARS. Then, 200 µL of extraction solution was transferred into each well of the 96‐well plate and the absorbance at 562 nm was measured using a luminometer (BIOTEK Synergy HTX).

### Alkaline Phosphatase (ALP) Staining and Quantification

After 14 days of osteogenic induction, the cells were washed with PBS followed by fixation with 4% paraformaldehyde for 30 min. ALP staining was performed using an Alkaline Phosphatase Test Kit (Beyotime, #P0321M). Briefly, the cells were incubated with the staining solution prepared according to the manufacturer's protocol for 15 min at 37 °C. The staining solution was discarded and the cells were washed three times with PBS and images of the cells were captured using a microscope. For the quantification of ALP, the cells were lysed with RIPA lysis buffer for 30 min at 4 °C and centrifuged at 11000×g for 30 min at 4°C. 50 µL of the working solution consisting of an equal volume of solution A and solution B from the staining kit was added to each well of a 96‐well plate, and 15 µL of the cell lysates supernatant was added. The mixture was then incubated for 15 min at 37 °C, and 75 µL of solution C was added to the mixture. The absorbance at 520 nm was measured using a luminometer (BIOTEK Synergy HTX). The results were normalized to the protein concentration in each well.

### Oil Red O (ORO) Staining

After 7 days of adipogenic induction, the cells were washed with PBS followed by fixation with 4% paraformaldehyde for 30 min. ORO staining was performed using an Oil Red O Staining Kit (Beyotime, #C0157S). Briefly, ORO staining solution was prepared by mixing ORO solution and diluent at a ratio of 3:2 followed by filtration through a 0.45 µm filter. The cells were stained with ORO staining solution at room temperature for 20 min. The cells were then washed three times with PBS, and images were captured using a microscope.

### Alcian Blue Staining

An Alcian Blue and Nuclear Fast Red Staining Kit (Beyotime, #C0155S) was used. After 25 days of chondrogenic induction, the cells were washed with PBS and stained with Alcian blue solution (pH 2.5) for 1 h. After three washes with PBS, the cells were stained with Nuclear Fast Red solution for 10 min. The cells were then washed with PBS and images were captured using a microscope.

### In Vivo Osteogenic Induction Assay

BMMSC transfected with KAT2A overexpression lentiviruses or control vectors in the absence of TNF‐*α* were cultured under osteogenic differentiation conditions for 7 days. Then, 5 × 10^5^ BMMSC were collected and transplanted on hydroxyapatite (HA)/tricalcium phosphate (TCP) for 24 h and transplanted into the subcutaneous dorsal space of 6‐ to 8‐week‐old BALB/c nu/nu mice. Osteogenic induction medium was locally injected every 3 days. The mice were euthanized 6 weeks after transplantation, and the grafts were collected for the follow‐up experiments.

### Isolation of Mouse Splenocytes and Naïve CD4+ T Cells

Mice were subjected to cervical dislocation following anesthesia with isoflurane. The mice were then sterilized in 70% ethanol for 5 min. The spleen was dissected by aseptic operation and homogenized. The crushed spleen tissue was resuspended in PBS and filtered through a 70‐µm cell strainer. Red blood cells (RBCs) were lysed by incubating the cells in RBC lysis buffer (Solarbio, #R1010) for 15 min at 4 °C. Splenocytes were then collected by centrifugation at 1500 rpm for 5 min. For the isolation of naïve CD4+ T cells in the spleen, a Mouse Naïve CD4+ T Cells Isolation Kit (Miltenyi Biotec, #130‐104‐453) was used according to the manufacturer's protocol. The obtained splenocytes were incubated with a cocktail of biotinylated negative selection antibodies and then subjected to magnetic labeling with anti‐biotin microbeads. The magnetically labeled cells were absorbed using a Separation Column in a magnetic field, and naïve CD4+ T cells were collected for subsequent experiments.

### Isolation of Human Peripheral Blood Mononuclear Cells (PBMCs) and Naïve CD4+ T Cells

Peripheral blood donated by six healthy volunteers was added to an equal volume of Ficoll (TBD, #LDS1075) and then subjected to density gradient centrifugation. The buffy coat was extracted, washed twice with PBS, and resuspended in 1640 medium (GIBCO, #C11875500BT). The collected PBMCs were then counted and seeded as indicated. For the isolation of naïve CD4+ T cells, a Naïve CD4+ T Cell Isolation Kit (Miltenyi Biotec, #130‐094‐131) was used. PBMCs were sequentially incubated with a cocktail of biotinylated negative selection antibodies and anti‐biotin microbeads. The magnetically labeled cells were deleted using a Separation Column in a magnetic field and, naïve CD4+ T cells were collected for subsequent experiments.

### Proliferation Assay

To measure the immunosuppressive capacity of human BMMSC, the isolated human PBMCs were incubated with 5 µm carboxyfluorescein succinimidyl ester (CFSE, Invitrogen, #C34570) for 10 min at 37 °C. FBS was then added, and the cells were incubated for 5 min. The cells were washed twice with PBS and counted. The CFSE‐labeled PBMCs were cocultured with BMMSC at a cell ratio of 1 BMMSC per 10 PBMCs in 1640 medium containing 10% FBS, 1 µg mL^−1^ anti‐human CD3/CD28 antibodies (PeproTech, #05121‐25, #10311‐25) and 100 IU mL^−1^ IL‐2 (Prosperich, #CSBSJ‐IL‐2). For the measurement of mouse BMMSC, the CFSE‐labeled splenocytes were cocultured with mouse BMMSC at a cell ratio of 1 BMMSC per ten splenocytes in 1640 medium containing 10% FBS, 1 µg mL^−1^ anti‐mouse CD3/CD28 antibodies (PeproTech, #05112‐25, #10312‐25) and 10 ng mL^−1^ IL‐2 (PeproTech, #212‐12). On the fourth day, the cells were collected, stained with live/dead dye 780, and detected by flow cytometry.

### Th17 Differentiation Inhibition Assay

Naïve CD4+ T cells from human and mouse were cocultured with human and mouse BMMSC at a ratio of 10:1, respectively. The cells were cultured in Th17 polarization conditions containing anti‐CD3/CD28 antibodies, IL‐2, 20 µg mL^−1^ IL‐23 (PeproTech, #200‐23, R&D, #1887‐ML), 10 µg mL^−1^ IL‐6 (PeproTech, #200‐06, R&D, #406‐ML) and 5 µg mL^−1^ TGF‐β1 (PeproTech, #100‐21C, R&D, #7666‐MB). The Th17 polarization conditions were refreshed on the fourth day. After 6 days of culture, the Cell Stimulation Cocktail (eBioscience, #00‐4975‐03) was added to the cells at a dilution of 1:500 and incubated for 6 h. The cells were then collected and stained with live/dead violet dye (BioLegend, #423113) for 15 min. After fixation for 20 min and permeabilization for 30 min with solution A and solution B from the FIX&PERM Kit (Invitrogen, #FAS004), respectively, the cells were incubated with anti‐IL‐17A‐APC (BD, #560437, BioLegend, #506916) for 30 min. The cells were then washed and analyzed by flow cytometry.

### Detection of Th17 Frequency In Vivo

Splenocytes were extracted as described above and incubated with the Cell Stimulation Cocktail at a dilution of 1:500 for 6 h. The cells were collected and stained with anti‐CD4‐PE (BioLegend, #100407) for 30 min. The cells were then stained with live/dead 780 dye, fixed, permeabilized, stained with anti‐IL‐17A‐BV421 (BioLegend, #512321), and analyzed by flow cytometry as described above.

### Micro‐CT Scanning

The harvested tissues were fixed with 4% paraformaldehyde for 24 h, and micro‐CT was performed using a Siemens Inveon CT scanner. Quantification of new bone formation was performed using an Inveon Research Workplace (Siemens). 3D CT images were reconstructed using RadiAnt DICOM Viewer software (v5.0.2).

### Histological Staining

The harvested samples were fixed with 4% paraformaldehyde for 24 h, decalcified with 50 nm EDTA solution for 2 weeks, and embedded in paraffin. The sections were deparaffinized and rehydrated by immersion in xylene and ethanol at decreasing concentrations.

For hematoxylin‐eosin (H&E) staining, the rehydrated sections were stained with hematoxylin (Servicebio, #G1004) for 10 min and then immersed in 1% alcoholic hydrochloric acid solution for 5 s. After washing with PBS, the sections were stained with eosin (BOSTER, #AR1180‐2) for 2 min. The sections were then washed, dehydrated, and sealed with neutral resin. Images were captured using a microscope. For the evaluation of arthritis inflammation infiltration, the H&E‐stained sections were scored according to the following criteria: 0, normal joint; 1, mild thickening of the lining layer or mild infiltration of the underlying layer; 2, mild thickening of the lining layer plus mild infiltration of the underlying layer; 3, moderate thickening of the lining layer, moderate infiltration of the underlying layer, and presence of cells in the synovial space; 4, severe infiltration of synovium with many inflammatory cells.

For safranin O‐fast green staining, a modified Saffron‐O and Fast Green Stain Kit (Solarbio, #G1371) was used. The rehydrated sections were stained with the Weigert staining solution of the kit for 5 min and then immersed in the acidic differentiation solution for 15 s. The sections were then stained with a fast green solution for 5 min and differentiated with a weak acidic solution for 10 s. The sections were dried, stained with safranin O solution for 5 min, dehydrated, and sealed with neutral resin. Images were then captured using a microscope. For the examination of cartilage damage, sections were scored based on safranin O‐fast green staining according to the following criteria: 0, normal joint; 1, erosion of part of the cartilage surface; 2, erosion of the cartilage surface and cartilage destruction; 3, cartilage erosion and destruction, and coverage with connective tissue.

For Masson staining, a Masson Trichrome Staining Kit (KeyGEN, #KGMST‐8004) was used. The rehydrated sections were sequentially stained with solutions A, B, C, and D for 10, 5, 1, and 5 min, respectively. The sections were then washed with PBS, dehydrated, and sealed with neutral resin. Images were captured using a microscope.

### Tissue Immunofluorescence Staining

The harvested samples were fixed with 4% paraformaldehyde for 24 h, decalcified with 50 nM EDTA solution for 2 weeks, and embedded in paraffin. The sections were deparaffinized, rehydrated, incubated in 0.5% Triton X‐100 solution for 30 min and then with 10 mm citrate buffer and microwaved at 750 W for 20 min for antigen retrieval. After returning to room temperature, the sections were incubated with 5% normal goat serum for antigen blockade. The sections were then incubated with the indicated primary antibodies at 4 °C overnight. On the second day, after washing with PBS, the sections were incubated with fluorescein‐labeled secondary antibodies for 1 h at room temperature away from light. Images were captured using a confocal imaging system (Nikon Eclipse Ni‐E). The following antibodies were used: anti‐Ki67 (Abcam, #ab279653), anti‐CD105 (Abcam, #ab221675), anti‐KAT2A (Santa Cruz, #sc‐365321), anti‐Osteocalcin (OCN) (Abcam, # ab93876), and anti‐GFP (CST, #2955). The following secondary antibodies were used: Alexa Fluor 488‐conjugated goat anti‐mouse IgG secondary antibody (Invitrogen, #A‐11001), Alexa Fluor® 488‐conjugated goat anti‐rabbit IgG secondary antibody (Invitrogen, #A‐11008), Alexa Fluor™ 555‐conjugated goat anti‐mouse IgG secondary antibody (Invitrogen, #A‐21422), and Alexa Fluor 555‐conjugated goat anti‐rabbit IgG secondary antibody (Invitrogen, #A‐21428).

### Tissue Immunohistochemical Staining

Sections of harvested samples were obtained as described above. The sections were deparaffinized, rehydrated, incubated in 10 mm citrate buffer, and microwaved at 750 W for 20 min for antigen retrieval. After returning to room temperature, the sections were incubated with 3% H_2_O_2_ for 25 min and incubated with 5% normal goat serum for 30 min and then with anti‐Collagen I (ABclonal, #A16891), anti‐Collagen II (ABclonal, #A1560) and anti‐Collagen X (Abcam, #ab260040) separately at 4 °C overnight. On the next day, the sections were incubated with secondary antibodies, and color development was performed using an SP Rabbit & Mouse HRP Kit (CWBIO, #CW2069S). Images were captured with a microscope.

### Statistical Analysis

The data from this study are presented as scatter plots with means and standard deviations. Statistical analysis was performed using GraphPad Prism 8.0 software (GraphPad Prism Software, CA, USA). The normality of the samples was tested with the Shapiro‐Wilk test. The differences between independent samples were analyzed with the two‐tailed Student's *t*‐test and paired t‐test for paired samples. The analysis of three or more groups was performed by one‐way ANOVA, followed by Bonferroni's post hoc comparisons. Two‐way repeated‐measures ANOVA was performed to analyze the differences in arthritis scores between the two groups. The sample sizes (n) for each statistical analysis were shown in figure legends. The *p‐*value cutoff was set as 0.05.

## Conflict of Interest

The authors declare no conflict of interest.

## Author Contributions

Z.‐p.S., J.‐t.L., and J.‐j.L. contributed equally to this work. H.‐y.S., Y.‐f.W. and Z.‐y.X. designed the study. Z.‐p.S. and J.‐t.L. performed the cell experiments. Z.‐p.S., J.‐j.L. and Z.‐k.L. performed the animal experiments. Z.‐p.S., Y.‐s.C. Z.‐q.Z. and P.‐t.X. performed the histological experiments. Z.‐p.S., J.‐j.L., Y.‐p.Z. and X.‐j.X. performed the bioinformatic analysis. Z.‐p.S., J.‐t.L., and Z.‐k.L analyzed and interpreted the data. G.Z., G.‐w.Y., and W.‐h.Y. provided technical support. Z.‐p.S., Z.‐y.X. and Y.‐f.W. wrote and reviewed the manuscript. The work was supervised by H.‐y.S.

## Supporting information

Supporting Information

## Data Availability

The data that support the findings of this study are available in the supplementary material of this article.
