# Supplementary material for: TNF‐α‐Induced KAT2A Impedes BMMSC Quiescence by Mediating Succinylation of the Mitophagy‐Related Protein VCP
Source: Adv Sci (Weinh). 2023 Dec 25;11(10):2303388. doi: 10.1002/advs.202303388 (PMC10933659; doi:10.1002/advs.202303388)
Supplement: Supplementary file 1 — Supporting Information [file ADVS-11-2303388-s001.pdf]

ADVANCED SCIENCE
Open Access

Supporting Information

for *Adv. Sci.*, DOI 10.1002/advs.202303388

TNF-α-Induced KAT2A Impedes BMMSC Quiescence by Mediating Succinylation of the Mitophagy-Related Protein VCP

Zepeng Su, Jinteng Li, Jiajie Lin, Zhikun Li, Yunshu Che, Zhaoqiang Zhang, Guan Zheng, Guiwen Ye, Wenhui Yu, Yipeng Zeng, Peitao Xu, Xiaojun Xu, Zhongyu Xie, Yanfeng Wu* and Huiyong Shen**

Supporting Information

TNF-α-induced KAT2A impedes BMMSC quiescence by mediating succinylation of the mitophagy-related protein VCP

Zepeng Su†, Jinteng Li†, Jiajie Lin†, Zhikun Li, Yunshu Che, Zhaoqiang Zhang, Guan Zheng, Guiwen Ye, Wenhui Yu, Yipeng Zeng, Peitao Xu, Xiaojun Xu, Zhongyu Xie, Yanfeng Wu*, Huiyong Shen**

†These authors contributed equally to this work.

The Supporting Information for this manuscript includes:

Figure S1. The phenotypes and differentiation potentials of BMMSC.

Figure S2. TNF-α concentrations above 100 ng/mL do not more significantly activate BMMSC.

Figure S3. Gating strategies used in the flow cytometry analysis of the activation of BMMSC in vivo.

Figure S4. Glycine promotes mitophagy and attenuates OXPHOS in BMMSC.

Figure S5. Succinylation of VCP K18 has no effect on mitophagy and the quiescence of BMMSC.

Figure S6. KAT2A has no effect on mitophagy and the quiescence of BMMSC expressing VCP-K658R.

Figure S7. Functional comparison of quiescent and activated BMMSC.

Table S1. Prediction of the binding of the domains of VCP to MFN1/2.

Table S2. Primer sequences used in this study.

The other Supporting Information for this manuscript includes:

Data file S1: Gene expression of BMMSC treated with or without TNF-α in the RNA-sequencing

Data file S2: Succinylation profiling data of BMMSC treated with or without TNF-α

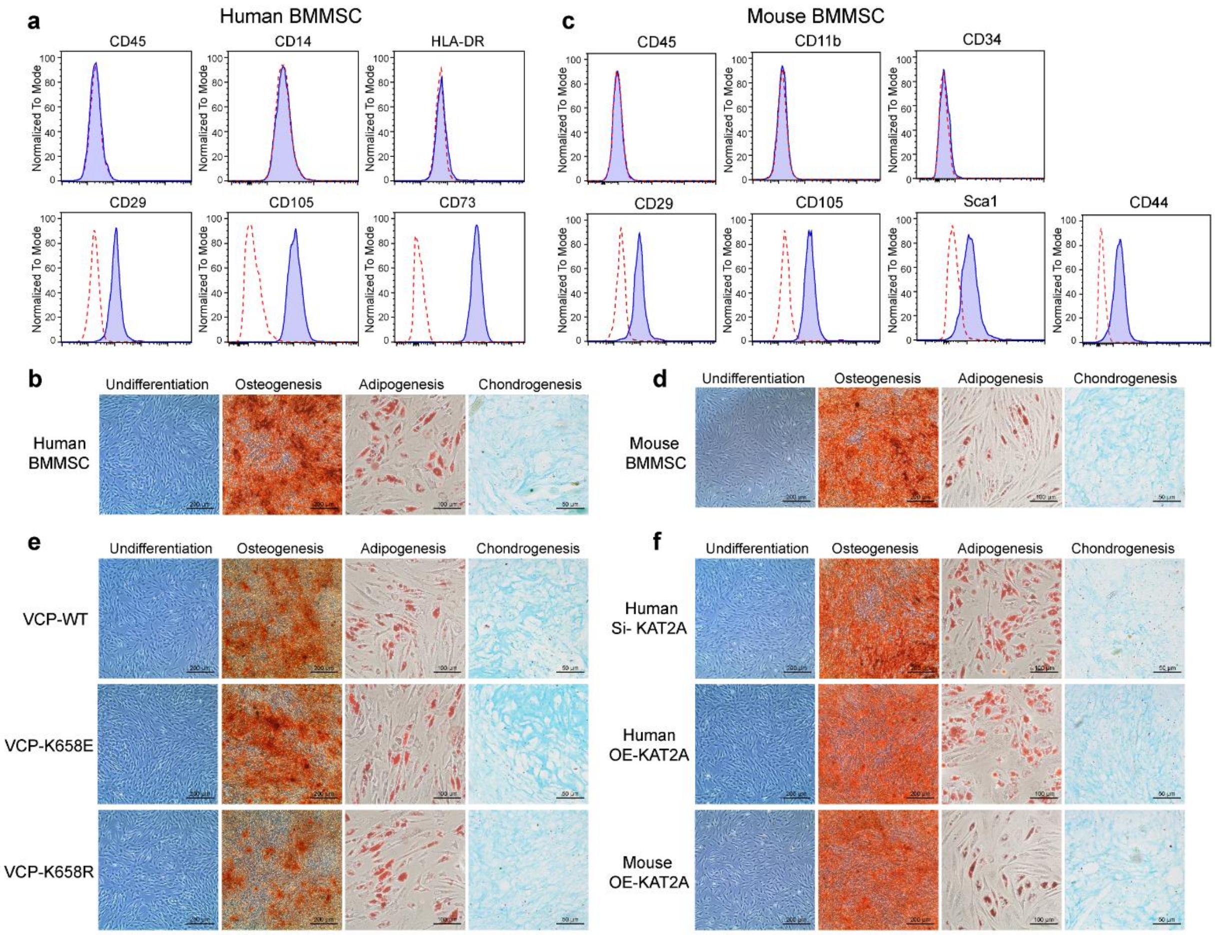

Figure S1. The phenotypes and differentiation potentials of BMMSC. a The phenotypes of human (h) BMMSC were identified by flow cytometry using negative surface markers CD45, CD14 and HLA-DR, and positive surface markers CD29, CD105 and CD73. **b** Osteogenic, adipogenic and chondrogenic differentiation potentials of hBMMSC. **c** The phenotypes of mouse (m) BMMSC were identified by flow cytometry using negative surface markers CD45, CD11b and CD34, and positive surface markers CD29, CD105, Sca1 and CD44. **d** Osteogenic, adipogenic and chondrogenic differentiation potentials of mBMMSC. **e** Osteogenic, adipogenic and chondrogenic differentiation potentials of hBMMSC modified by VCP-Wt, VCP-K658E and VCP-K658R. **f** Osteogenic, adipogenic and chondrogenic differentiation potentials of hBMMSC modified by OE-KAT2A and Si-KAT2A, and mBMMSC modified by OE-KAT2A.

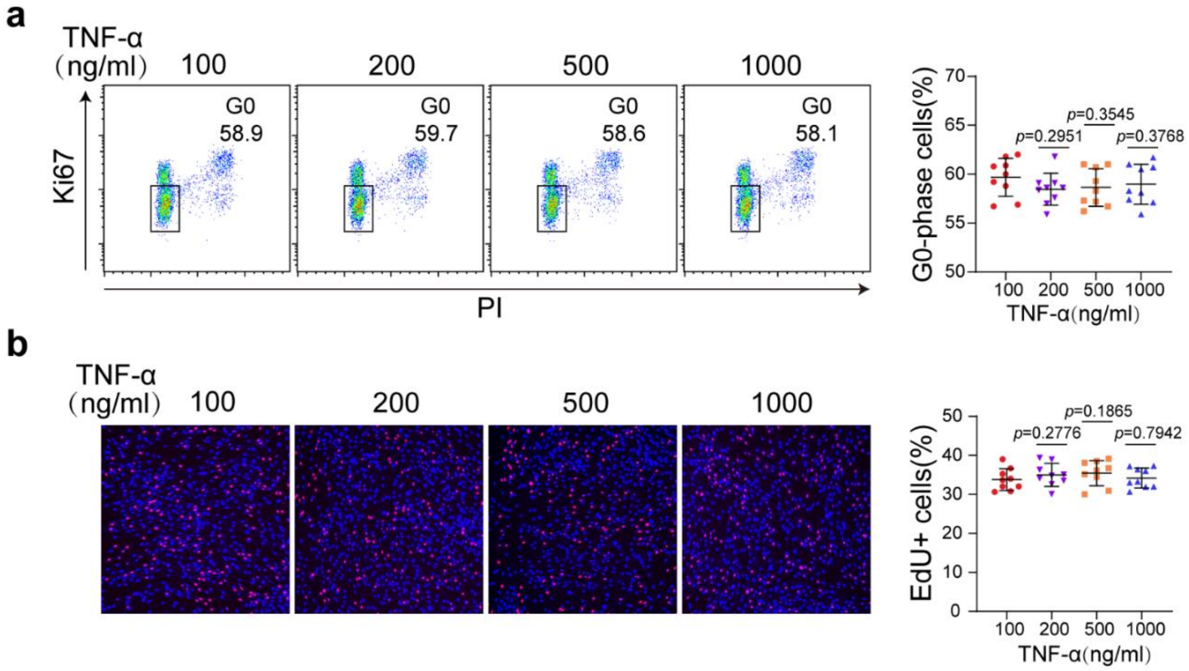

Figure S2. TNF-α concentrations above 100 ng/mL do not more significantly activate BMMSC. a Flow cytometry analysis showed no difference in the proportion of G0-phase cells among hBMMSC treated with 100, 200, 500 and 1000 ng/mL TNF-α (n=9). **b** EdU assay showed no difference in the proportion of EdU+ cells among hBMMSC treated with 100, 200, 500 and 1000 ng/mL TNF-α (n=9). The values are presented as the mean±SD. The statistical analyses were performed with one-way ANOVA.

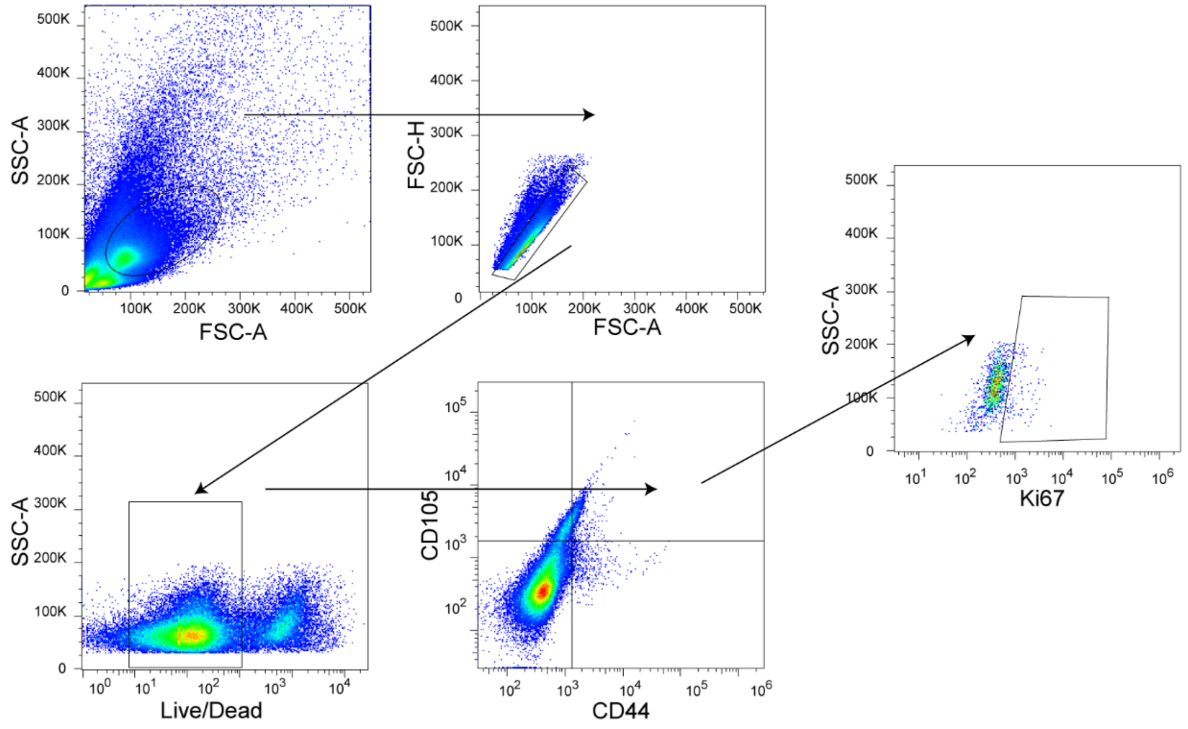

Figure S3. Gating strategies used in the flow cytometry analysis of the activation of BMMSC in vivo.

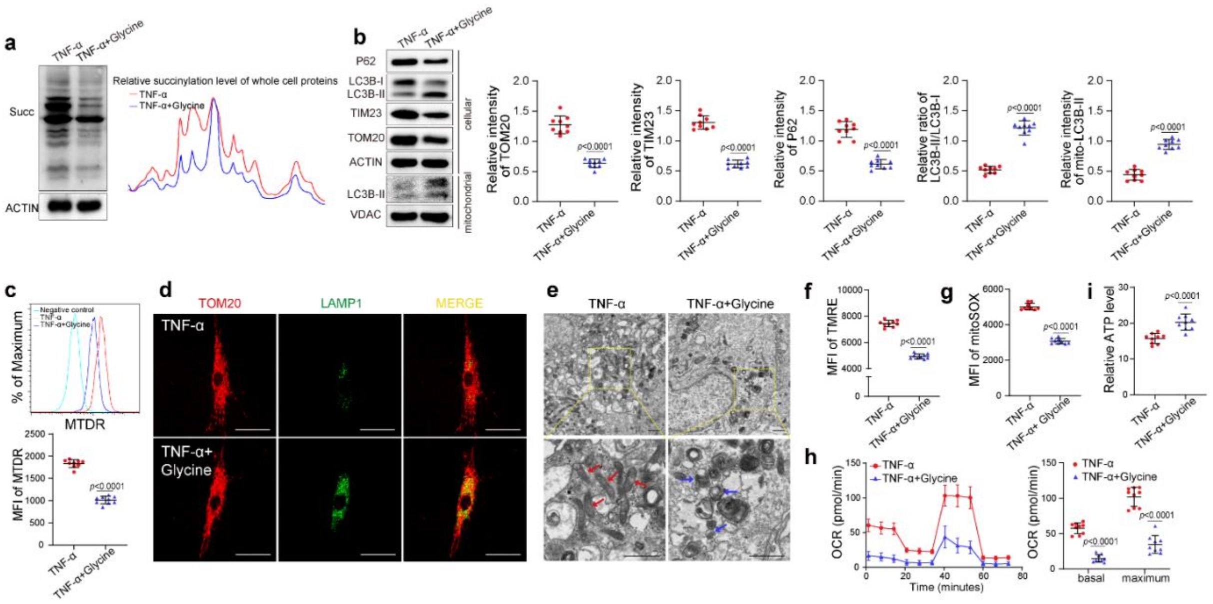

Figure S4. Glycine promotes mitophagy and attenuates OXPHOS in BMMSC. a Glycine downregulated the protein succinylation level in hBMMSC (n=9) in the presence of TNF-α. **b** Glycine decreased the protein levels of TOM20, TIM23 and P62 and increased the ratio of LC3B-II/LC3B-I and the level of mitochondria-related LC3B-II in hBMMSC (n=9) in the presence of TNF-α. **c** Glycine reduced the MFI of MTDR in hBMMSC (n=9) in the presence of TNF-α. **d** Glycine promoted the colocalization of mitochondria and lysosomes in the presence of TNF-α. Scale bar=50 μm. **e** Glycine increased the number of fragmented mitochondria and mitochondria engulfed by autophagosomes in the presence of TNF-α. Scale bar=2 μm. **f-i** Glycine reduced the TMRE MFI (**f**), MitoSOX MFI (**g**) and the OCR (**h**), and elevated the ATP level (**i**) in hBMMSC (n=9) in the presence of TNF-α. The values are presented as the mean±SD. The statistical analyses were performed with a two-tailed paired t-test.

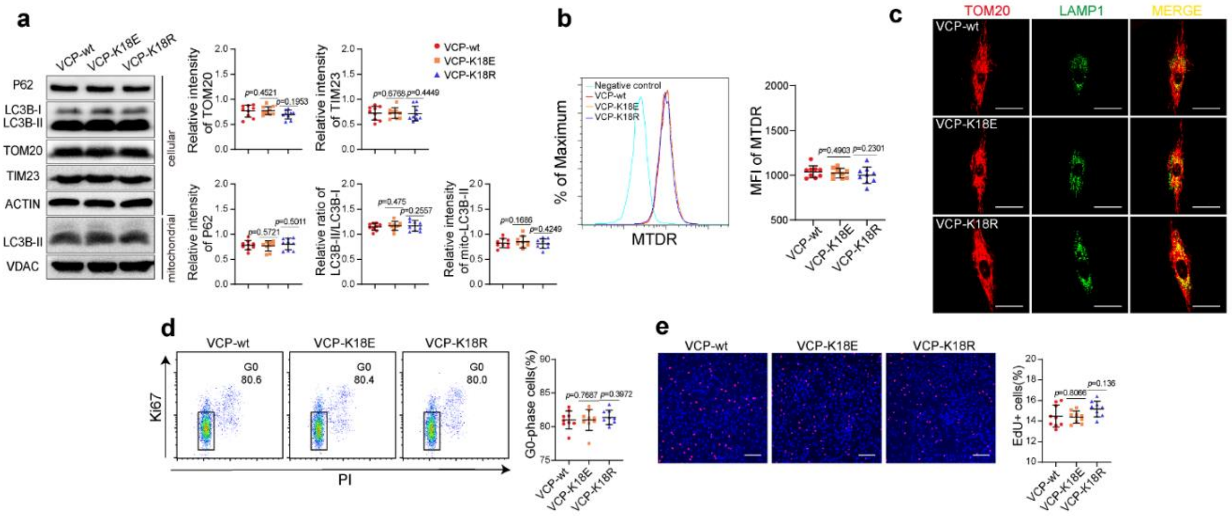

Figure S5. Succinylation of VCP K18 has no effect on mitophagy and the quiescence of BMMSC. **a** VCP-K18E or VCP-K18R did not alter the protein levels of P62, TOM20 and TIM23, the ratio of LC3B-II/LC3B-I and the level of mitochondria-related LC3B-II compared with VCP-Wt (n=9). **b** Compared with VCP-Wt, VCP-K18E or VCP-K18R had no effect on the MFI of MTDR (n=9). **c** Compared with VCP-Wt, VCP-K18E or VCP-K18R had no effect on the colocalization of mitochondria and lysosomes. Scale bar=50 μm. **d, e** Compared with VCP-Wt, VCP-K18E or VCP-K18R had no effect on the proportions of G0-phase (**d**) or EdU+ (**e**) hBMMSC (n=9). Scale bar=200 μm. The values are presented as the mean±SD. The statistical analyses were performed with one-way ANOVA followed by Bonferroni’s post hoc comparisons.

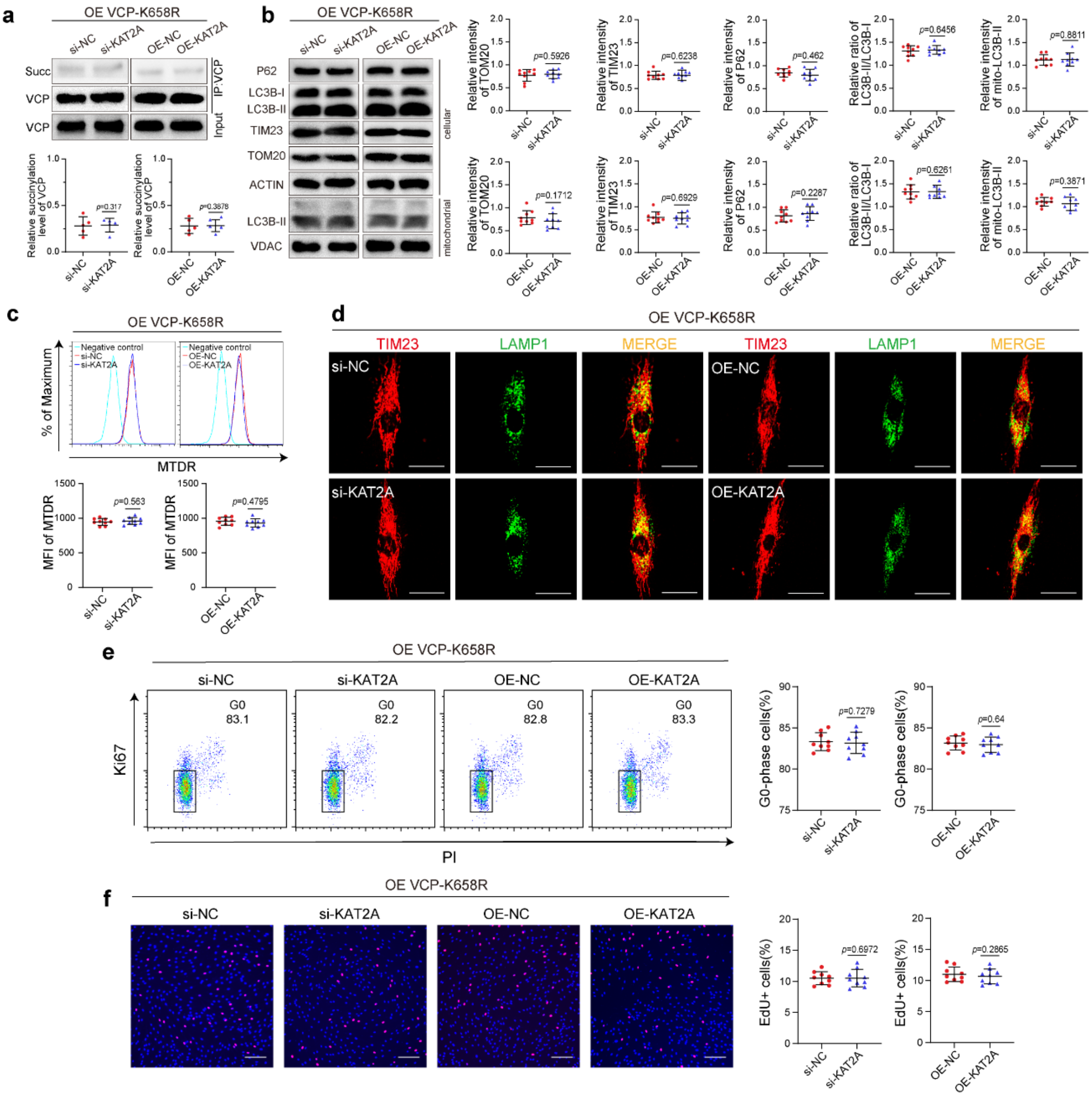

Figure S6. KAT2A has no effect on mitophagy and the quiescence of BMMSC expressing VCP-K658R. a KD/with TNF-α or OE/without TNF-α did not impact the succinylation level of VCP in hBMMSC expressing VCP-K658R (n=5). **b** KD/with TNF-α or OE/without TNF-α had no effect on the protein levels of P62, TOM20 and TIM23, the ratio of LC3B-II/LC3B-I or the level of mitochondria-related LC3B-II in hBMMSC expressing VCP-K658R (n=9). **c** KD/with TNF-α or OE/without TNF-α had no effect on the MFI of MTDR in hBMMSC expressing VCP-K658R (n=9). **d** KD/with TNF-α or OE/without TNF-α had no effect on the colocalization of mitochondria and lysosomes in hBMMSC expressing VCP-K658R. **e, f** KD/with TNF-α or OE/without TNF-α had no effect on the G0-phase (**e**) and EdU+ (**f**) proportion in hBMMSC expressing VCP-K658R (n=9). Scale bar=200 μm. The values are presented as the mean±SD. The statistical analyses were performed with a two-tailed paired t-test.

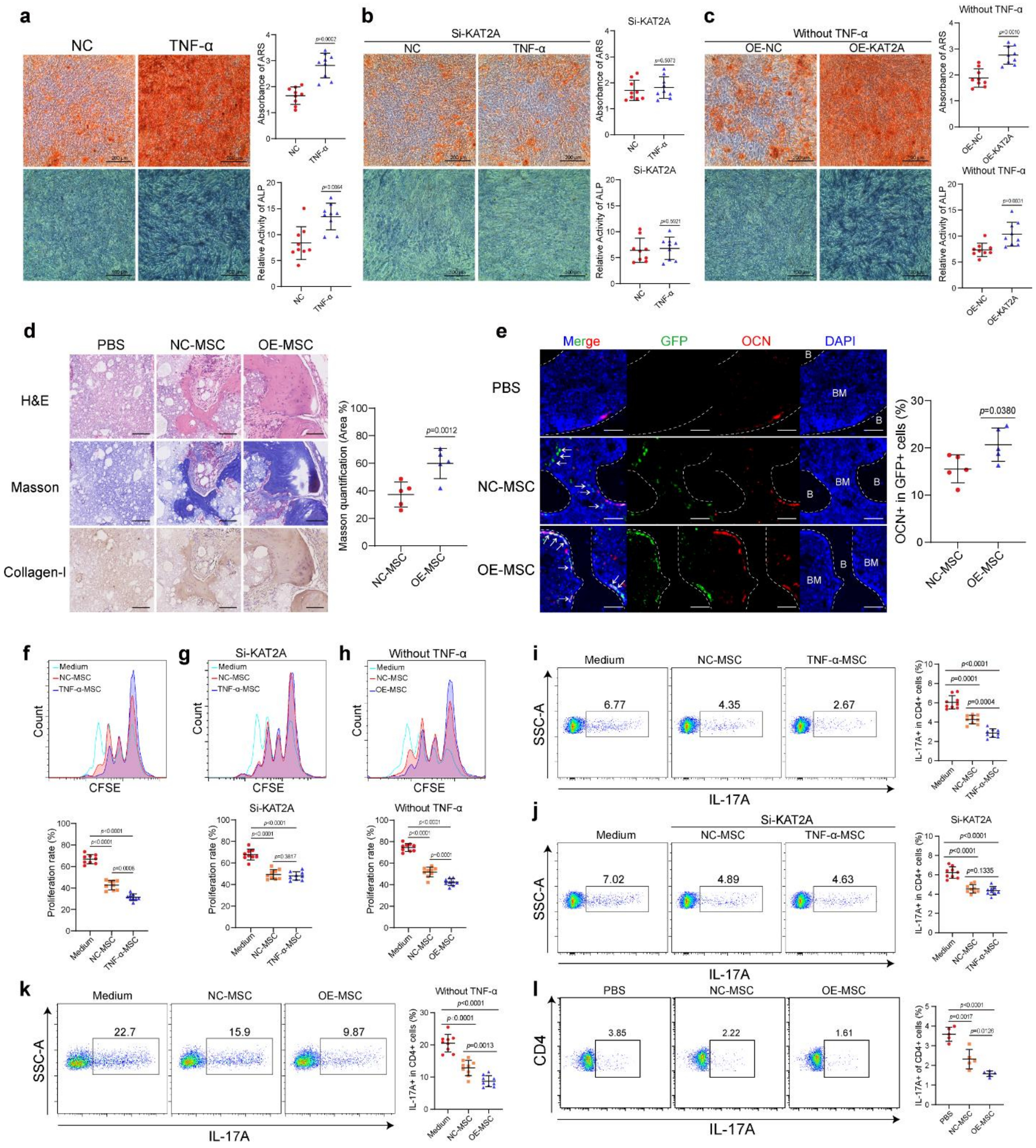

Figure S7. Functional comparison of quiescent and activated BMMSC. a TNF-α-activated hBMMSC exhibited enhanced ARS and ALP staining compared with quiescent hBMMSC (n=9). **b** The enhanced ARS and ALP staining by TNF-α-induced activation were blocked in hBMMSC with KAT2A knockdown (n=9). **c** Mouse OE-MSC exhibited enhanced ARS and ALP staining compared with NC-MSC (n=9). **d** H&E, Masson and Collagen-I staining revealed that mouse OE-MSC formed a larger osteogenesis area than NC-MSC on HA/TCP transplanted in vivo (n=5). **e** The expression of OCN in mouse OE-MSC was higher than NC-MSC 14 days after transplantation (n=5). The dotted line represents the demarcation between the bone marrow and trabeculae, the arrows indicate BMMSC, B indicates bone and BM indicates bone marrow. Scale bar=50 μm. **f** TNF-α-activated hBMMSC exhibited a stronger suppressive effect on PBMCs proliferation than quiescent hBMMSC (n=9). **g** The enhanced suppressive effect on PBMCs proliferation by TNF-α-induced activation was blocked in hBMMSC with KAT2A knockdown (n=9). **h** Mouse OE-MSC exerted an enhanced suppressive effect on PBMCs proliferation compared with NC-MSC (n=9). **i** TNF-α-activated

hBMMSC exhibited stronger suppressive effect on Th17 differentiation than quiescent hBMMSC (n=9). **j** The enhanced suppressive effect on Th17 differentiation by TNF-α-induced activation was blocked in hBMMSC with KAT2A knockdown (n=9). **k** Mouse OE-MSC exerted an enhanced suppressive effect on Th17 differentiation compared with NC-MSC (n=9)**. l** Mouse OE-MSC more prominently reduced Th17 frequency in CD4+ T cells in the spleen of CIA mice than NC-MSC (n=5). The values are presented as the mean±SD. The statistical analyses were performed as follows: one-way ANOVA followed by Bonferroni's post hoc comparisons (f-l), two-tailed Student's t-test (d, e) and two-tailed paired t-test (a-c).

Table S1. Prediction of the binding of the domains of VCP to MFN1/2

	N-terminal Domain	D1 ATPase Domain	D2 ATPase Domain
MFN1	△G=-8.31 kcal/mol<br>Kd=8.03e-07 M	△G= -9.69 kcal/mol<br>Kd=7.83e-08 M	△G=-11.05 kcal/mol<br>Kd=7.93e-09 M
MFN2	△G=-7.72 kcal/mol<br>Kd=2.20e-06 M	△G= -9.09 kcal/mol<br>Kd=2.14e-07 M	△G= -10.45 kcal/mol<br>Kd=2.17e-08 M

Table S2. Primer sequences used in this study

Gene	Forward primer (5'-3')	Reverse primer (3'-5')
ACTIN	CATGTACGTTGCTATCCAGGC	CTCCTTAATGTCACGCACGAT
CCNB1	AATAAGGCGAAGATCAACATGGC	TTTGTTACCAATGTCCCCAAGAG
CCNA2	CGCTGGCGGTACTGAAGTC	GAGGAACGGTGACATGCTCAT
CDK1	AAACTACAGGTCAAGTGGTAGCC	TCCTGCATAAGCACATCCTGA
CDK2	CCAGGAGTTACTTCTATGCCTGA	TTCATCCAGGGGAGGTACAAC
P16	GATCCAGGTGGGTAGAAGGTC	CCCCTGCAAACTTCGTCCT
P21	TGTCCGTCAGAACCCATGC	AAAGTCGAAGTTCCATCGCTC
P27	AACGTGCGAGTGTCTAACGG	CCCTCTAGGGGTTTGTGATTCT
